# Targeted Therapy Modulates the Secretome of Cancer-Associated Fibroblasts to Induce Resistance in HER2-Positive Breast Cancer

**DOI:** 10.3390/ijms222413297

**Published:** 2021-12-10

**Authors:** Melani Luque, Marta Sanz-Álvarez, Andrea Santamaría, Sandra Zazo, Ion Cristóbal, Lorena de la Fuente, Pablo Mínguez, Pilar Eroles, Ana Rovira, Joan Albanell, Juan Madoz-Gúrpide, Federico Rojo

**Affiliations:** 1 Department of Pathology, Fundación Jiménez Díaz University Hospital Health Research Institute (IIS-FJD, UAM)—CIBERONC, 28040 Madrid, Spain; melani.luque@quironsalud.es (M.L.); marta.sanza@quironsalud.es (M.S.-Á.); andreasantamaria_95@hotmail.com (A.S.); szazo@fjd.es (S.Z.); 2 Translational Oncology Division, OncoHealth Institute, Health Research Institute-Fundación Jiménez Díaz (IIS-FJD, UAM), 28040 Madrid, Spain; ion.cristobal@quironsalud.es; 3 Genetics Department, Health Research Institute-Fundación Jiménez Díaz (IIS-FJD, UAM), Center for Biomedical Network Research on Rare Diseases (CIBERER), ISCIII, 28040 Madrid, Spain; lorena.fuente@quironsalud.es (L.d.l.F.); pablominguez@gmail.com (P.M.); 4 Institute of Health Research INCLIVA-CIBERONC, 46010 Valencia, Spain; Pilar.Eroles@uv.es; 5 Department of Physiology, University of Valencia, 46010 Valencia, Spain; 6 Cancer Research Program, IMIM (Hospital del Mar Research Institute), 08003 Barcelona, Spain; arovira@imim.es (A.R.); 96087@parcdesalutmar.cat (J.A.); 7 Medical Oncology Department, Hospital del Mar-CIBERONC, 08003 Barcelona, Spain; 8 Department of Experimental and Health Sciences, Faculty of Medicine, Universitat Pompeu Fabra, 08002 Barcelona, Spain

**Keywords:** breast cancer, HER2-positive, tumour microenvironment, targeted therapy, trastuzumab, resistance, cancer-associated fibroblast, label-free proteomics, miRNA

## Abstract

The combination of trastuzumab plus pertuzumab plus docetaxel as a first-line therapy in patients with HER2-positive metastatic breast cancer has provided significant clinical benefits compared to trastuzumab plus docetaxel alone. However, despite the therapeutic success of existing therapies targeting HER2, tumours invariably relapse. Therefore, there is an urgent need to improve our understanding of the mechanisms governing resistance, so that specific therapeutic strategies can be developed to provide improved efficacy. It is well known that the tumour microenvironment (TME) has a significant impact on cancer behaviour. Cancer-associated fibroblasts (CAFs) are essential components of the tumour stroma that have been linked to acquired therapeutic resistance and poor prognosis in breast cancer. For this reason, it would be of interest to identify novel biomarkers in the tumour stroma that could emerge as therapeutic targets for the modulation of resistant phenotypes. Conditioned medium experiments carried out in our laboratory with CAFs derived from HER2-positive patients showed a significant capacity to promote resistance to trastuzumab plus pertuzumab therapies in two HER2-positive breast cancer cell lines (BCCLs), even in the presence of docetaxel. In order to elucidate the components of the CAF-conditioned medium that may be relevant in the promotion of BCCL resistance, we implemented a multiomics strategy to identify cytokines, transcription factors, kinases and miRNAs in the secretome that have specific targets in cancer cells. The combination of cytokine arrays, label-free LC-MS/MS quantification and miRNA analysis to explore the secretome of CAFs under treatment conditions revealed several up- and downregulated candidates. We discuss the potential role of some of the most interesting candidates in generating resistance in HER2-positive breast cancer.

## 1. Introduction

Breast cancer is the most common cancer in women, representing about 25–30% of all cancer cases worldwide, and it is the leading cause of cancer death in women aged 20 to 59 years [1]. Breast cancer is a heterogeneous disease that comprises multiple subtypes with different morphological and clinical features and distinct outcomes [2]. Among these subtypes, breast cancer with amplification and/or overexpression of the human epidermal growth factor receptor 2 (HER2, ErbB2) accounts for about 20% of all human breast cancers [3]. Amplification of HER2 in breast cancer patients correlates with disease progression and poor survival outcome and disease recurrence [3,4]. Targeting the HER2 receptor has become an attractive therapeutic approach in treating HER2-positive breast cancer patients. Trastuzumab (Herceptin) and pertuzumab (Perjeta) are the first-line drugs targeting HER2 [5]. They are recombinant humanised monoclonal antibodies directed against different extracellular regions of HER2. As they act through different but complementary mechanisms of action, their combined use in clinical practice provides a more comprehensive blockade of HER2 signalling, as well as a more effective therapeutic strategy than treating patients with a single HER2 monoclonal antibody [6,7]. Based on the results from the phase III CLEOPATRA trial, trastuzumab combined with pertuzumab plus docetaxel was approved as the first-line treatment of HER2-positive metastatic breast cancer and remains the standard of care for this indication [8,9].

Despite the clinical benefit obtained from the above therapies, patients eventually progress due to acquired resistance, so there is an urgent need for alternative treatments [6,10]. Many different mechanisms have been described that can lead to acquired resistance in cancer, including genetic and epigenetic changes in cancer cells [11]. Additionally, growing evidence indicates that the tumour microenvironment (TME) also mediates resistance in solid tumours and has a significant impact on therapeutic response and clinical outcome [12,13]. The tumour stroma includes the extracellular matrix (ECM), immune and inflammatory cells, endothelial cells and fibroblasts, among others [14]. Activated fibroblasts that are found in association with cancer cells, known as cancer-associated fibroblasts (CAFs), are a source of growth factors, cytokines and ECM proteins, promoting proliferation and survival of tumour cells [14,15]. Furthermore, recent work has demonstrated that CAFs contribute to drug resistance acquisition in cancer cells, suggesting that they could be targeted in patients with cancer, including breast cancer patients [16,17,18]. In breast carcinomas, CAFs represent about 80% of the fibroblasts surrounding cancer cells [19]. Numerous studies suggest that CAFs play an important role in resistance to endocrine therapy, chemotherapy and targeted therapies [16,17,20]. However, the mechanisms that CAFs deploy in the acquisition of resistance to targeted therapies in HER2-positive breast cancer remains unknown [21,22].

In normal tissues, fibroblasts are the cells responsible for facilitating repair and regeneration during wound healing and tissue inflammation. Consistent with the saying that cancer is “a wound that never heals”, CAFs play a key role in promoting tumourigenesis and contributing to the malignant phenotype. The way in which CAFs perform this conditioning of tumour cells and other cells of the TME is through their production of ECM and many proinflammatory growth factors and cytokines. This is a unique feature of CAFs, and therefore the identification of CAF-secreted proteins (collectively known as the CAF secretome) is crucial to elucidate the underlying mechanisms governing CAF-mediated drug resistance in cancer cells. The secretion of major cytokines by CAFs, such as interleukins (IL-6, IL-8 and IL-10), transforming growth factor-β (TGF-β), CXC chemokine ligands (CXCL12 and CXCL14) and vascular endothelial growth factor (VEGF) promotes the recruitment of immunosuppressive cells into the TME, driving a chronic inflammatory, proangiogenic and immunosuppressive intratumoural environment. In addition, the ability of CAFs to impact cancer cells has also been attributed to CAF-derived proteins [23]. Mass spectrometry-based secretome analysis is a powerful tool used to identify and characterise secreted proteins and has been widely employed in the study of CAF secretome (for an extensive review see [24]). On the other hand, strong evidence has emphasised the relevance of microRNAs (miRNAs) in cancer hallmarks, such as tumourigenesis, metastasis and chemoresistance [25]. Furthermore, the potential role of CAF-derived miRNAs has also been described in breast cancer [26,27]. Hence, the miRNA profile of the CAF secretome could also be an interesting source of potential candidates that may be involved in drug resistance acquisition. To this end, we initially characterised the ability of CAFs to modulate resistance to treatment in HER2-positive breast cancer cell lines, using cellular assays of proliferation, migration, spheroid formation and molecular characterisation of EMT markers. We then explored which cytokines played a significant role in this modulation using antibody microarrays. Subsequently, we performed a differential expression study of secretome proteins by label-free mass spectrometry. Finally, an analysis of miRNA profiling by NGS provided us with information, at the transcriptional level of potential resistance-modulating molecules.

As is common in many types of cancer and in different processes leading to tumourigenesis and subsequent tumour expansion, acquired resistance to treatment sometimes involves multiple signalling cascades. In the case of CAFs, our interest in analysing the molecular components of the secretome went beyond the dynamic description of the usual markers (αSMA, FAP, S100A4, CV1, PDGFRβ, IL6, etc.), and we aimed to characterize the pathophysiological role of CAFs in the context of their interaction with tumour cells in the TME, analysing possible activations of EGFR, TGF-β, JAK/STAT, Wnt/β-catenin and Hippo signalling pathways in relation to anti-HER2 resistance. Based on a cellular model of interaction between HER2-positive breast cancer cell lines and CAFs from patients of the same tumour type, we aimed to identify, using a multiomics-based analysis, those candidate cytokines, proteins and miRNAs from the CAF secretome that may contribute to the development of resistance to first-line HER2-targeted therapy (trastuzumab plus pertuzumab plus docetaxel) in the tumour cell. We believe that our work provides useful information about certain effector and regulatory molecules, signalling pathways and biological processes that, in the context of the interaction in the TME between the tumour cell and CAFs, could be relevant as inducers of the resistance that the HER2-positive breast cancer cell develops towards HER2-targeted therapy.

## 2. Results

### 2.1. CM[CAF-200/TPD] Promotes Resistance to Anti-HER2 Therapies in HER2-Positive Breast Cancer Cell Lines

**Figure 1:**
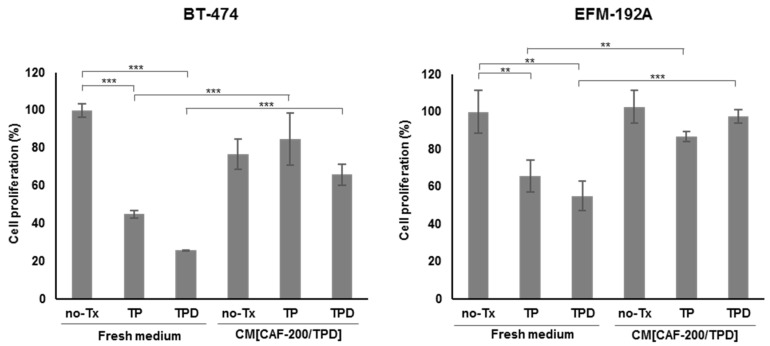
CM[CAF-200/TPD] induced acquired resistance to anti-HER2 therapy, even in presence of docetaxel-based chemotherapy. Effect of the addition of CM[CAF-200/TPD] on BT-474 and EMF-192A proliferation, after treatment with 15 μg/mL trastuzumab (T) plus 20 μg/mL pertuzumab (P), in combination with or without 0.5 nM docetaxel (D), for 7 days. No-Tx: no treatment. *n* = 3. ** denotes *p*-value < 0.01, *** denotes *p*-value < 0.001.

In order to examine the potential role of CAF-200 in the acquisition of resistance to anti-HER2 therapy by tumour cells, we measured the effect that secretions of CAFs into the CM generated on HER2-positive breast cancer, under TPD treatment conditions. We performed cell proliferation assays in two cell lines for 7 days (Figure 1). As expected, cells treated with trastuzumab and pertuzumab were initially sensitive to the drug combination. Interestingly, however, sensitivity to anti-HER2 therapy was significantly reduced when cells were exposed to CM[CAF-200/TPD] (Figure 1). Notably, both cell lines significantly reduced their sensitivity to therapy even in the presence of docetaxel (*p*-value < 0.001) (Figure 1). These results suggest that CAF-200 can induce resistance to anti-HER2 therapies through soluble factors secreted to the extracellular medium.

### 2.2. Increased Expression of Epithelial–Mesenchymal Transition-Related Markers in Breast Cancer Cell Lines Was Induced by CM[CAF-200/TPD]

**Figure 2:**
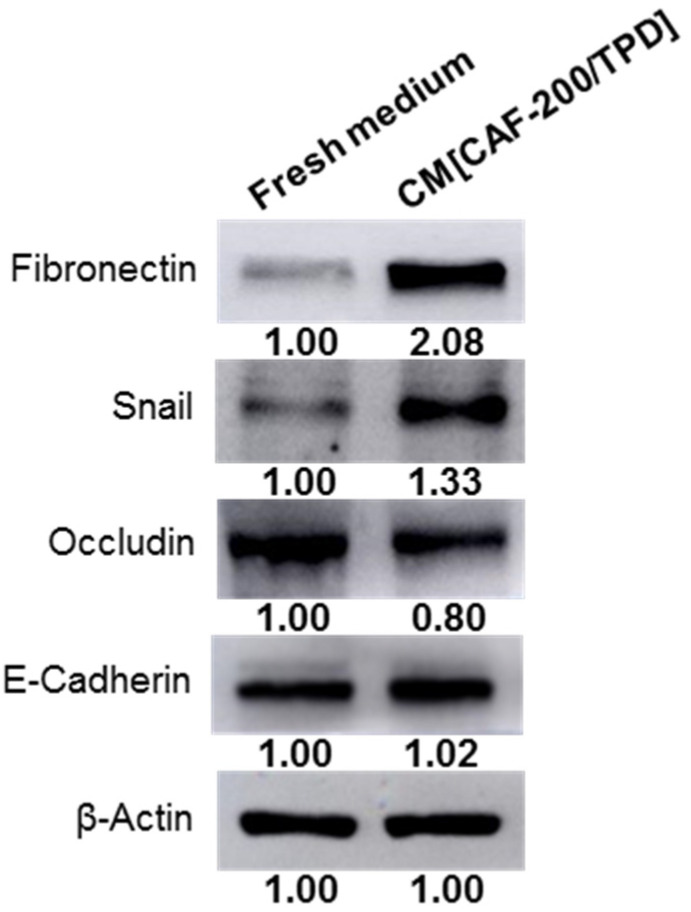
CM[CAF-200/TPD] induced greater expression of mesenchymal markers in BT-474 cancer cell line. WB analysis of EMT-related markers (fibronectin, Snail, occludin and E-cadherin) when BT-474 cells were cultured for 72 h under basal or CM conditions. Representative images of WB assays are depicted (*n* = 3). Relative abundance levels of protein up- or downregulation were determined by densitometric analysis of the images.

Although ample evidence suggests that CAFs play a crucial role in drug resistance in cancer, details about the mechanisms involved remain unclear. To investigate whether CAFs may induce Epithelial–Mesenchymal Transition (EMT)—a process that endows cells with a resistant phenotype—a WB analysis of fibronectin, Snail, occludin and E-cadherin was performed. The study showed an upregulation of mesenchymal markers (fibronectin and Snail) in BT-474 cells treated with CM[CAF-200/TPD] (Figure 2). Conversely, the expression levels of epithelial markers were not altered (E-cadherin) or were slightly downregulated (occludin) when cells were treated, as compared to untreated cells. These data suggest that CAF-200 may be involved in the promotion of EMT processes.

### 2.3. Changes in the Phosphorylation Pattern of HER2 and Downstream Signalling in Response to Anti-HER2 Therapies Was Induced by CM[CAF-200/TPD]

**Figure 3:**
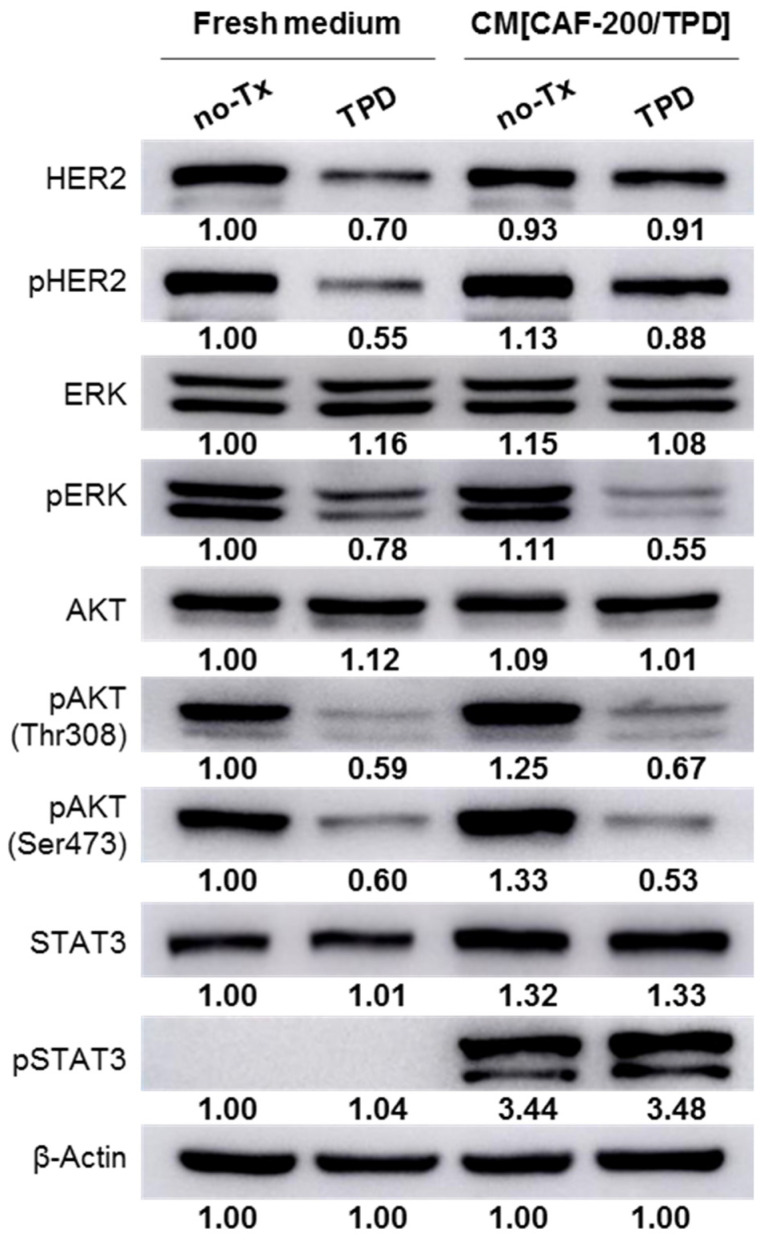
After treatment with CM[CAF-200/TPD], BT-474 cells showed changes in phosphorylation markers of cell division. BT-474 cells were treated for 72 h with TPD, either in fresh culture medium or CM[CAF-200/TPD]. Analysis of phosphorylation pattern of HER2 and other downstream proteins was assessed by WB. Representative images of three replicates are depicted. Relative abundance levels of protein up- or downregulation were determined by densitometric analysis of the images.

The WB analysis revealed no significant modification in the basal HER2 expression level of BT-474 cells cultured in CM[CAF-200/TPD] as compared to those cultured in a fresh culture medium. Similarly, HER2 phosphorylation levels did not change either (Figure 3). By contrast, and as expected, both receptor expression and phosphorylation levels were markedly reduced after treatment with TPD in a fresh medium. Interestingly, when cells were cultured in CM[CAF-200/TPD], TPD treatment induced a smaller decrease in HER2 phosphorylation levels compared to the control scenario (Figure 3). In contrast, the addition of CM[CAF-200/TPD] to BT-474 cells increased the intensity of the response to TPD in terms of ERK phosphorylation. At the level of AKT activation, hardly any difference was seen in either of the two phosphorylation residues studied. Most relevant to this analysis was the remarkable increase in the level of Signal Transducer and Activator of Transcription 3 (STAT3) phosphorylation (active) when cells were cultured in a medium containing CM[CAF-200/TPD], regardless of whether they were then treated with TPD or not. According to the literature, STAT3 signalling has been related to progression and poor response in breast cancer, and in particular to trastuzumab resistance in HER2-positive breast cancer [28].

### 2.4. CAFs Induced a Spheroid-Forming Phenotype in BT-474 Cells Treated with Anti-HER2 Therapies Plus Chemotherapy

**Figure 4:**
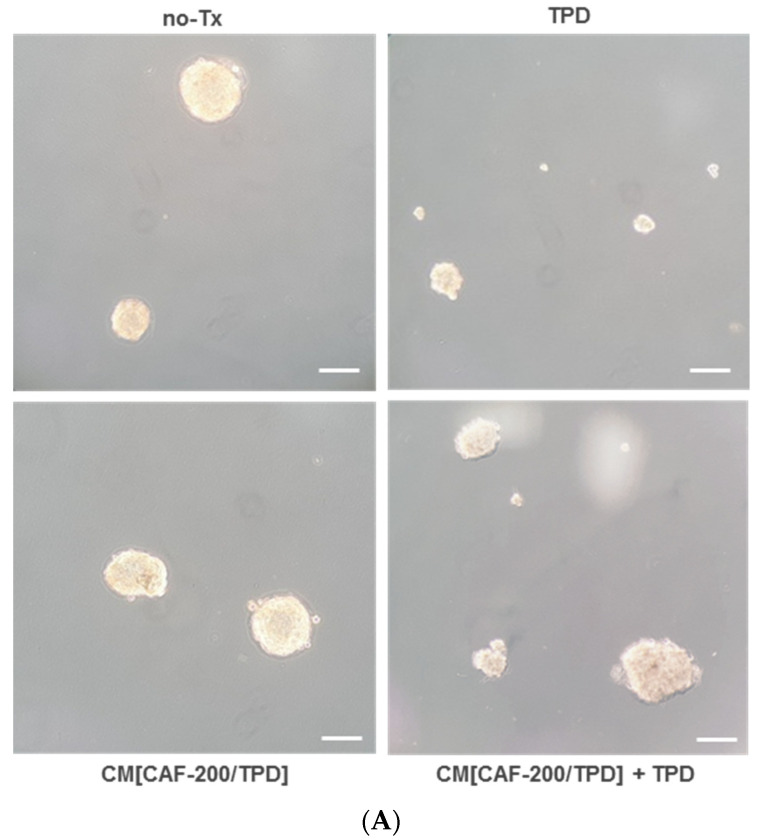

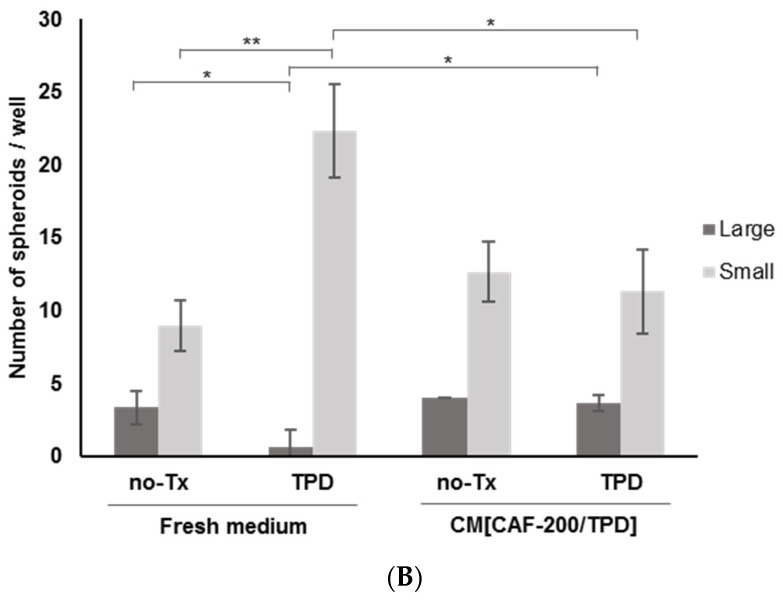
CM[CAF-200/TPD] induced changes in spheroid formation in BT-474 cells. Effect of CM[CAF-200/TPD] on spheroid formation in BT-474 cells, treated with or without TPD for 7 days. (**A**) Representative images of tumour spheroids (magnification: 5×). Scale bar: 100 μm. (**B**) Number of spheroids at day 7. Two types of spheroids were distinguished by their diameter: large and small. *n* = 3. *: *p*-value < 0.05; **: *p*-value < 0.01.

Spheroid assays have been proposed for the enrichment and characterisation of cancer stem cells (CSCs) or related cells, as CSCs have the ability to grow into spheres under anchorage-independent conditions. We therefore used this approach to examine the potential of CAF-200 to enrich the stem phenotype BT-474 cell population in the context of a response to TPD therapy. BT-474 cells were able to aggregate and form three-dimensional (3-D) spheroids on ultra-low-adhesion plates, where the adhesion to the well surface is very limited (Figure 4A). Depending on the size of the spheroids, we distinguished those whose diameter was <50 µm (termed “small, S”) or ≥150–200 µm (“large, L”). The treatment of the cells with TPD resulted in a marked change in the spheroid-forming capacity of each type, leading to an increase in the number of small spheroids and a decrease in the number of large spheroids (Figure 4B). Under a CM[CAF-200/TPD] culture, we observed an increase in the total number of spheroids under basal conditions, with no variation in the large/small ratio. However, the response of BT-474 cells to TPD in the presence of CM[CAF-200/TPD] was very different: there was little variation in the number of spheroids of either size, so that the number and ratio of large and small spheroids reflected a situation similar to that of BT-474 cells under basal conditions. These data may suggest a protective role of CM[CAF-200/TPD] in BT-474 cells against therapy, at least with regard to stemness-related features.

### 2.5. Tumour Cell Migration Increased in the Presence of the Molecular Milieu Secreted by CAFs

**Figure 5:**
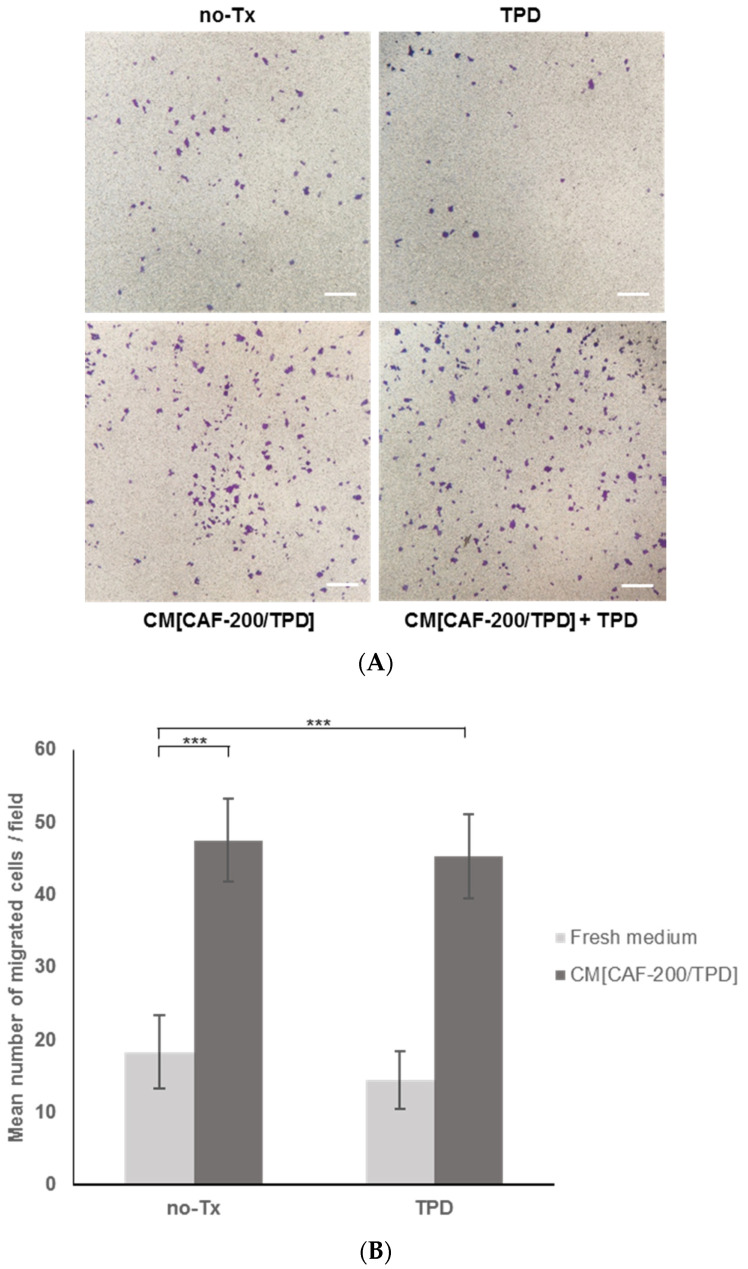
CM[CAF-200/TPD] stimulated transwell migration of BT-474 cells. Effect of CM[CAF-200/TPD] on migration capacity of BT-474 cells treated with TPD. (**A**) Representative images of migrated cells (magnification: 5×). Scale bar: 100 µm. (**B**) The number of migrated cells increased with CM[CAF-200/TPD] addition as compared to untreated cells in a fresh medium. Ten randomised fields were counted (magnification: 20×). *n* = 3. ***: *p*-value < 0.001.

CAFs are described to be, within EMT, the major contributors to tumour cell migration and invasion. Therefore, we performed BT-474 cell invasion assays to investigate whether CM[CAF-200/TPD] might have the ability to promote HER2-positive cell migration. We analysed the invasiveness of tumour cells in the presence or absence of CM[CAF-200/TPD] after 24 h by staining migrated cells with crystal violet to count them (Figure 5A). Interestingly, we observed a significant increase in migration in BT-474 cells when treated with CM[CAF-200/TPD] compared to untreated cells (Figure 5B), confirming the key role of CAFs in migration and invasion processes, through their paracrine signalling. Of note, this response of the cells to signal secretion from CAF-200 hardly varied under TPD treatment conditions. In summary, the invasive capacity of BT-474 cells was significantly increased by the addition of CM[CAF-200/TPD], even in the presence of anti-HER2 therapy combined with chemotherapy.

### 2.6. TME-Infiltrating miRNA-199b Could Be a Potential Target to Modulate Anti-HER2 Resistance in HER2-Positive BCCLs

MiRNAs are another key component of the CAF secretome, the dysregulation of which modulates the function of the tumour microenvironment. However, most studies have focused on investigating the functions of miRNAs within the tumour cells themselves, so there are still gaps in our understanding of how miRNAs function in the tumour microenvironment. We performed miRNA sequencing of the CAF-200 secretome comparing the miRNA expression profile after TPD treatment vs. pretreatment. A limma analysis was then performed, and miRNAs with a *p*-value < 0.05 were considered significant. Table 1 lists the miRNAs that displayed FC > 1.5. MiR-130a-3p showed the strongest relative upregulation (logFC = 1.96, *p*-value = 0.03). Interestingly, it has been previously related to cancer progression and recurrence in breast cancer. MiR-199b was also found to be upregulated in our CAF-200 secretome. Notably, miR-199b has also been described as being involved in breast cancer but, more importantly, the HER2 receptor has been revealed as its direct target [29]. Regarding some differentially downregulated miRNAs, miR-4787 was the most strongly downregulated (logFC = −2.19, *p*-value = 0.02) (Table 1). MiR-23b was also found to be downregulated in the CAF-200 secretome. This finding is contrary to some publications that have described miR-23b as an oncogenic miRNA in luminal breast cancer [30]. Validation and functional studies would therefore be necessary to adequately characterise its role in our model.

MiRNA-199b was one of the most upregulated candidates in our miRNA analysis of the CAF-200 secretome. It has been suggested as a possible target for HER2-positive breast cancer, as its role in inhibiting HER2 downstream signalling has been demonstrated in vitro [29]. Therefore, to assess its potential involvement in the acquisition of resistance to TPD therapy, we proceeded to transfect BT-474 cells with pre-miR-199b and then performed a proliferation assay. Under initial basal conditions, in the absence of treatment, cells transfected with pre-miR-199b showed a higher proliferation rate compared to those transfected with the negative control. However, a subsequent combined treatment abolished the proliferative advantage of cells transfected with pre-miR-199b to levels only slightly higher than the control (data not shown). Given the complexity of the secretome composition, it is likely that the resistance-inducing effect of anti-HER2 therapy does not depend on a single isolated factor, and therefore joint candidate validation assays may be required.

### 2.7. Cytokine Secretion from CAF-200 Was Modified by Combined Treatment with TPD

**Figure 6:**
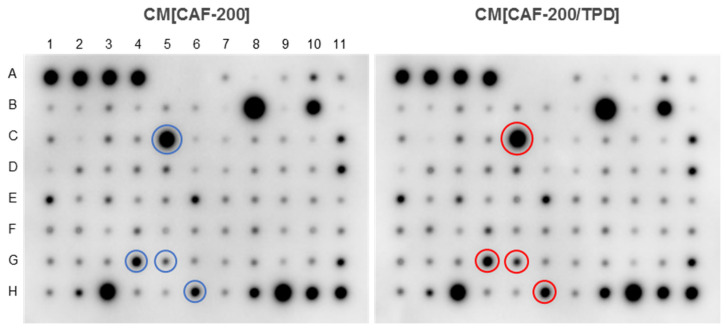
The secretion of several cytokines from CAF-200 was increased after treatment with TPD. CAF-200 cells were cultured and treated for 72 h with TPD. Then, cells were cultured in serum-free conditions for 24 h, and the CM was analysed using a Human Cytokine Array C5 (*n* = 3). Representative images of arrays incubated with CM[CAF-200] or CM[CAF-200/TPD] are shown. The localisation of some of the most relevant overexpressed cytokines is indicated by coloured circles, and their identities correspond to: CCL2 (C5), CXCL10 (G4), LIF (G5) and TGFB2 (H6).

CAFs play an essential role in promoting tumour initiation, progression, metastasis and therapeutic resistance. Through the secretion of cytokines and chemokines, they mediate tumour-promoting inflammation and generate a potent immunosuppressive intratumoural environment. So, we decided to scrutinise the cytokine secretion of CAF-200 cells in order to find out whether the induction of resistance to anti-HER2 therapy in tumour cells was related to cytokine production by CAFs under TPD treatment situations. The 80 analysed cytokines are listed in Table S1. Globally, the initial visual impression of anticytokine antibody arrays incubated with the CAF-200 secretome (either in the absence or presence of TPD drugs) was of intense signals for a few elements and faint changes in the intensity of most of the spots for different experimental conditions (Figure 6). From our experience with protein microarrays, this is probably because a chemiluminescence-developed blot device offers a relatively small dynamic range of signal intensity [31].

The signal from some factors was certainly of high magnitude, as expected (Table 2). For example, some interleukins (IL-6, IL-8), chemokines (CCL2, CXCL10) and ECM regulators (TIMP1, TIMP2, TGFB2) showed high intensities, suggesting that CAFs were expressing them at high levels even under basal conditions. An analysis of cytokines differentially secreted according to treatment condition revealed that some of these same factors showed a modulation in their expression when the CAFs were treated with TPD: some were upregulated (CCL2, CXCL10, TGFB2, LIF), while others were downregulated (angiogenin, OPN). In addition, other factors with lower expression levels were also upregulated by treatment, either positively (FGF-7, FGF-6) or negatively (IL-7, CCL22, IGF1, TNFA, CCL15). Since many cytokines exert their molecular effect at low doses, it is also important to note that some factors that were expressed at low levels under basal conditions nevertheless showed remarkable variations in their abundance levels when CAF-200s were treated with TPD. This is the case, for example, of IL-13, FGF-7, TPO (increase) and IL-7, CCL22, IL-5 (decrease).

We finally selected 26 cytokines, according to differential expression and fold-change parameters, as the most relevantly different in the treated CAFs. To place the role of treatment-activated cytokines in the context of specific biological processes, we identified the GO categories to which these cytokines are assigned. We ran the GO analysis on cellular components (CC), biologic processes (BP) and molecular function (MF) (Table S2). In addition to confirming that all 26 cytokines are secreted into the extracellular space by CAFs, a cellular localisation analysis indicates the organelles of production and processing (endoplasmic reticulum and Golgi), as well as the means of secretion to the exterior (granules, vesicles and exosomes). Interestingly, most of the cytokines activated by treatment are described as being associated with cellular regulatory processes, either metabolic regulation or general catalytic regulation through hydrolysis. In addition, many of these molecules are involved in regulating immune system signalling, both in the macrophage response and in the chemotaxis of natural killer cells, lymphocytes and eosinophils. It is also noteworthy that half of these cytokines play roles in regulating the Epithelial–Mesenchymal transition. The GO analysis of molecular functions showed that a large percentage of the candidates were involved in inflammatory responses, neutrophil and lymphocyte migration and the positive regulation of ERK1 and ERK2 cascades. To have a more comprehensive view of which molecular processes could be affected in the tumour cell by the secretion of these cytokines, we performed a functional enrichment analysis that allowed us to identify those signalling pathways potentially altered as a consequence of TPD treatment in CAF-200. Thus, beyond the expected signalling due to the activation of different cytokine groups, we discovered potential interactions in the vascular wall, inflammatory response and stemness activators and in an oncogenic context, signalling in the MAPK and mTOR pathways (Table S3). These findings agree with the roles already described for CAFs in tumour niches, where they contribute, through paracrine secretions, to activate protumourigenic pathways, contribute to EMT, maintain stemness in cancer stem cells, encourage the growth of new blood vessels and modulate inflammation and the immune response in the TME, all of which can eventually provide therapeutic resistance to the tumour cells.

### 2.8. Proteomic Analysis of CAF Secretome after Treatment Revealed Differentially Expressed Proteins

In this study, proteomic differences between CM[CAF-200] and CM[CAF-200/TPD] were investigated by LC–MS/MS. A label-free strategy was used in an attempt to comprehensively identify possible secreted targets that could be involved in anti-HER2 resistance. We compared whole CM[CAF-200] at baseline with CM[CAF-200/TPD] treated for 72 h. This analysis resulted in the identification of 1420 differentially expressed proteins (DEP), 1352 of which could be quantified. A total of 283 proteins displayed a q-value < 0.05, 145 of which had a variability percentage less than 30%. Of those, 96 proteins were upregulated in the treated CAF secretome as compared to the untreated, while 49 proteins were downregulated.

The PCA showed a good grouping of the replicates according to their treatment conditions for proteins with significant statistics (Figure S1). The Volcano plot revealed several proteins in both arms of the analysis showing high relative abundance levels for each differential experimental condition (Figure S2). Most of the proteins identified corresponded to the drug-treated secretome condition, as evidenced by the slight deviation from the mean, which is shifted towards the higher ratio values.

### 2.9. Gene Ontology and Functional Enrichment Analysis of CAF-200 Secretome Highlighted Oncogenic Processes and Regulation of Immune Response

The biological processes and molecular functions in which the 145 proteins selected are involved were deduced by a GO overrepresentation analysis (Figure S3). The first thing we observed was that about a quarter of the proteins were associated with multimolecular complexes (membrane receptors, proteasome and transcription complexes, etc.). In turn, these proteins were mostly associated with cellular processes (organisation of components, communication, response to stimuli and signal transduction), metabolic processes and regulators of biological activities. More specifically, the most significant processes were those associated with the regulation of transcriptional activities, metabolic functions and cellular homeostasis. This coincides with the description of the molecular functions associated with these secreted proteins, which are mostly interpreted in terms of binding to and the chaperoning of proteins, nucleic acids and macromolecular complexes; catalytic activity and the regulation of intracellular chemical reactions; regulators of enzymes, receptors and transcription; and the formation and stabilisation of molecular structures.

The list of the 145 differentially expressed proteins was subjected to GSEA to gain insight into the potentially relevant processes in which the CAF-200 secretome may be involved (Table S4). Depending on the collection of gene sets we used in each particular analysis, about a hundred gene sets with a *p*-value < 0.05 were identified. These sets may represent molecular pathways, ontological categories, expression/repression profiles, spatial clusters and immunological conditions, etc. Interestingly, some of the most relevant pathways were associated with the oncogenic processes involved in increased cell proliferation, cell growth and cell survival, such as PI3K/AKT/mTOR, NF-κB and RAS/MAPK pathways. The molecular signature of genes upregulated in breast lobular carcinoma was indicative of the genes involved in EMT, such as TGF-ß and Wnt signalling. We also observed gene sets with immunological functions associated with tumour invasion models. Finally, a pathway associated with genes upregulated in lung tissue EMT was also enriched.

**Figure 7:**
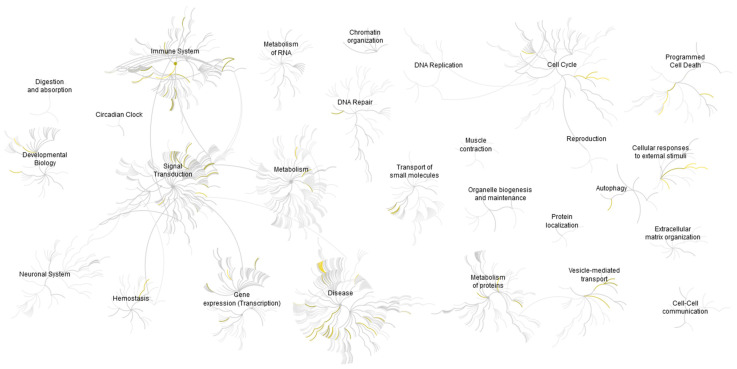
Genome-wide overview of the results of Reactome pathway analysis. Pathways are arranged in a hierarchy. The centre of each of the circular “bursts” is the root of a higher level track, e.g., “Immune system”. Each step away from the centre represents the next lower level in the track hierarchy. The colour coding denotes the overrepresentation of that track in its input dataset: yellow means overrepresentation, while light grey means that tracks are not significantly overrepresented.

A pathway overrepresentation analysis was performed by using the Reactome tools and database, which allowed us to visualise potential pathways and reactions activated in tumoural cells as a consequence of the effects of selected proteins and small molecules present in the CAF-200 secretome (Figure 7). Thus, we mostly confirmed the suggestions previously seen: regulation of metabolism, activation of signalling cascades initiated by membrane receptors (EGFR, FGFR and CCKR), modulation of signalling pathways involved in cell proliferation and survival, angiogenesis and invasion (RAS/MAPK, PI3K, VEGF and p53), as well as, most notably, the regulation of inflammation and immune response (Table 3). In addition, the enrichment in resistance reactions of the ERBB2 mutants to trastuzumab and other drugs is noteworthy.

### 2.10. Clinical Significance of the Different Protein Groupings

**Figure 8:**
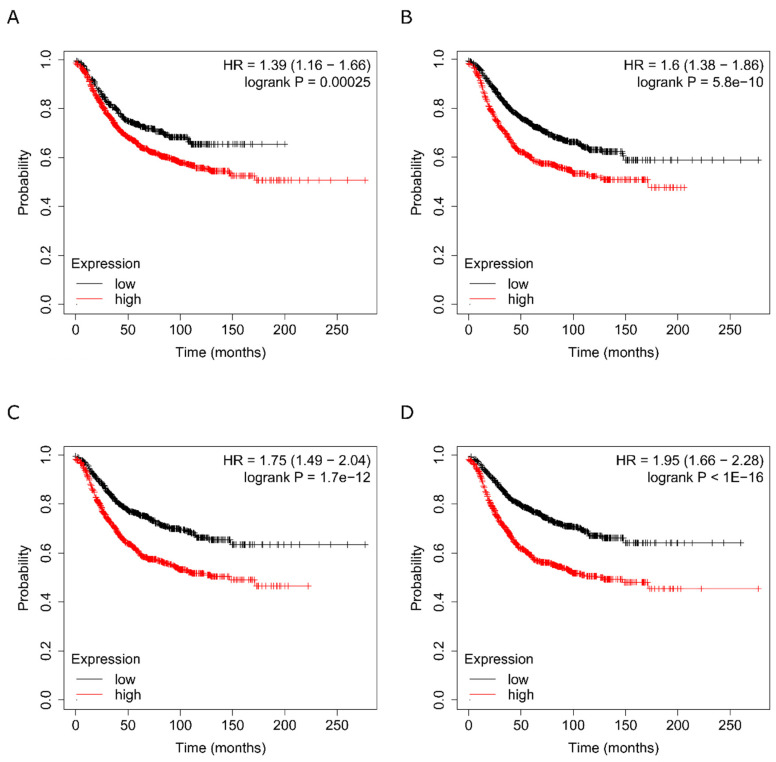
Associations of each classified group with clinical outcome. Clinical significance of molecular signatures assembled with (**A**) cytokines selected by antibody arrays, (**B**) proteins selected from MS–MS discovery assays in the CAF-200 secretome, (**C**) proteins associated in the functional enrichment analysis with neutrophil degranulation and (**D**) proteins related to immune system. Kaplan–Meier curves showing associations of expression levels of proteins with RFS in breast cancer are presented.

The effects that TME cells, and in particular CAFs, exert on tumour cells are mediated by cytokines and growth factors as well as by broad-spectrum proteins secreted into the environment. All these molecules play a more or less relevant role depending on their abundance, their co-expression with some other factor, the temporal dynamics of their expression, their localisation and their collaboration with other molecules within a signalling pathway or in a node. To discover the association of the gene expression levels of our identified proteins with clinical outcome, we used the Kaplan–Meier Plotter online survival analysis tool [32], which has massive gene expression data and survival information derived from more than 15,000 patients from databases including The Cancer Genome Atlas (TCGA), the Gene Expression Omnibus (GEO) and the European Genome-phenome Archive (EGA). Based on data obtained from previous analyses of cytokine antibody arrays and the MS-MS label-free study of the CAF-200 secretome, a meta-analysis was performed to determine the potential validity of the selected candidates as prognostic markers. We analysed the effect on survival of candidate proteins grouped in different batches according to the abundance ratio, *p*-value, q-value and fold change criteria. We assessed the relevance of the mean expression levels of various proteins in each group on relapse-free survival (RFS) in breast cancer in general and in the HER2-positive subtype in particular (Figure 8). Our results showed a distinct correlation for each group with the survival time. For example, the subset of the most significant cytokines (*p*-value < 0.05) discriminated those cases with a shorter RFS (HR = 1.39, Figure 8A), although their specific value did not add more relevance to the data obtained for the total secretome cytokines (Figure S4A). Similarly, in the group of 145 selected proteins, higher mean expression levels of the 96 proteins with positive abundance ratios (proteins overexpressed in the treated CAFs) were associated with a decreased RFS (HR = 1.61, Figure 8B). Conversely, in the group with inverse abundance ratios (higher expression in the non-treated CAFs), the lower abundance levels of the selected 49 proteins correlated with a reduced RFS (Figure S4B). Interestingly, when we selected groups of proteins by ontological or functional categories, we found that some biological functions, or particular physiological conditions or certain signalling pathways also discriminated (in some cases with even greater statistical significance) overexpression conditions that were associated with elevated RFS rates. For example, when we analysed some of the pathways highlighted in Table 3, we found that an overabundance of proteins involved in neutrophil degranulation correlated very significantly with a lower RFS (HR = 1.75, Figure 8C), and furthermore, the group of immune response-associated proteins was associated with a twofold HR for those with overexpression (HR = 1.95, Figure 8D). In the context of our model of TME-mediated cellular resistance to anti-HER2 treatment, these data suggest that intercellular communication within the tumour mass may be playing a role in contributing to the acquisition of such resistance. These data suggest that, by refining the analysis methodology, prognostically useful information could be extracted from the proteins secreted into the extracellular milieu, in order to obtain indicators of the tumour microenvironment under conditions of therapeutic exposure. These complex molecular signatures could help discriminate patients for whom specific treatments directed against these targets and pathways could make a significant difference in the survival prognosis.

### 2.11. Suggested Role of the Resistance-Inducing Secretome in Drug Sensitivity and Resistance in HER2-Positive Breast Cancer Cell Lines

The perturbation that a treatment causes in cells always results in the modulation of the expression of a gene of interest, which makes it possible to monitor the downstream consequences. However, with the exception of very few cases studied, there is no way to systematically determine the cellular effects of a given compound: these alterations in the regulatory mechanisms of genes and proteins often lead to unexpected off-target activities, influencing drug sensitivity, sometimes generating resistance to treatment and ultimately resulting in side effects that limit clinical use [33]. To investigate whether our molecular signatures could be used to predict therapeutic efficacy in response to certain drugs used in breast cancer, we decided to integrate the transcriptome data representing the mRNA expression from the CCLE with either trastuzumab sensitivity traits from our own assays [34] and from others [35] or docetaxel response signature from the GDSC database. No data were available for pertuzumab.

**Figure 9:**
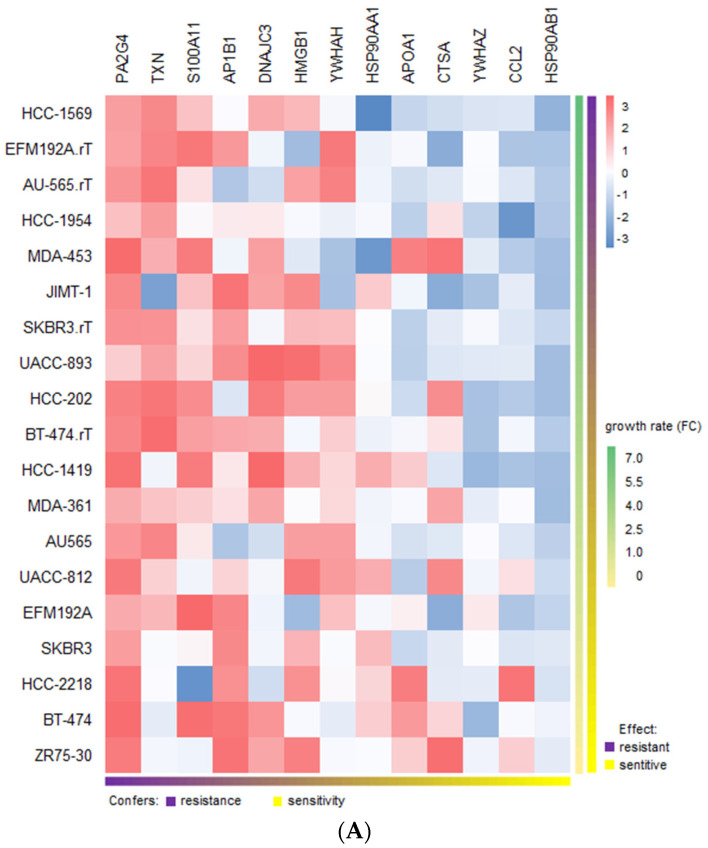

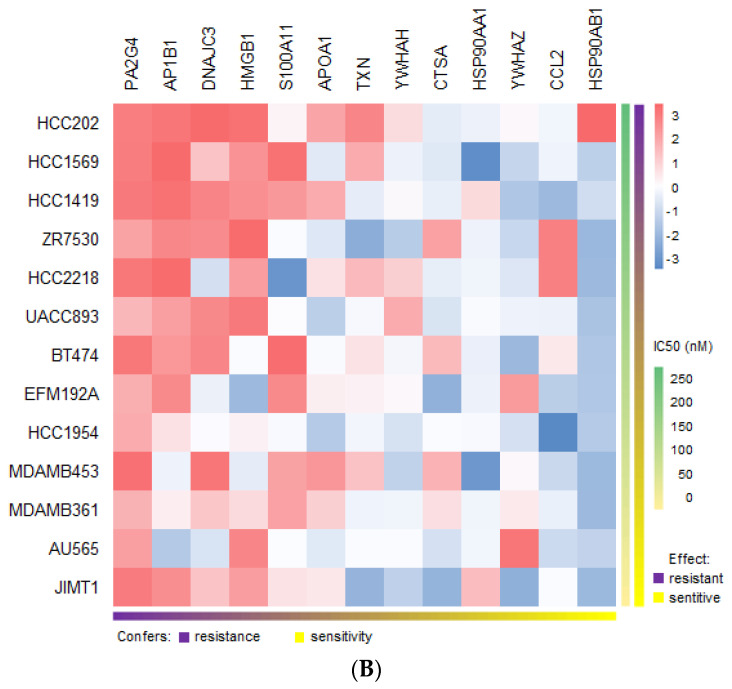
Heatmaps for the associations of the immune response signature with drug sensitivity and resistance in breast cancer. (**A**) Resistance/sensitivity to trastuzumab in a panel of HER2-positive BCCLs as a function of the gene expression values of the selected proteins. (**B**) Id. for docetaxel.

We retrieved the expression values from CCLE for the group of proteins that mark for immune response and calculated the linear regression against the sensitivity values for each drug in a panel of 20 HER2-positive breast cancer cell lines (ANOVA *p*-value < 0.01). In the case of trastuzumab, since the effect of the drug is mainly cytostatic but not cytotoxic, it is usually not meaningful to determine its IC50 and therefore no such data are available in the literature. Alternatively, we had sensitivity/resistance values for trastuzumab determined by proliferation assays (growth rate FC) both in trastuzumab-sensitive and trastuzumab-resistant cell lines [34,35]. Our analysis revealed potential links between some of the proteins and resistance in HER2-positive breast cell lines (Figure 9A). The two proteins that showed the highest correlation with trastuzumab resistance were *PA2G4* (EBP1) and TXN. This is interesting, as the two proteins have been described to be involved respectively in ErbB2 regulation and trastuzumab resistance.

With respect to docetaxel, IC50 values from GDSC were employed. In this case (Figure 9B), we observed that some of the identified proteins/genes had already been correlated with resistance, such as *PA2G4* and thioredoxin (TXN) in breast cancer [36], high-mobility group box 1 (HMGB1) in several cancer types [37], S100A11 in prostate cancer [38] and heat shock proteins and chemokine (C-C motif) ligand 2 (CCL2) in multiple contexts. Other candidates, such as AP1B1, APOA1, YWHAH and YWHAZ, however, do not appear to be associated with docetaxel resistance in cancer. Although this is a preliminary estimate, as the number of genes and cell lines is modest, this analysis reveals some molecular features that could be relevant when considering the potential usefulness in assessing the therapeutic response.

## 3. Discussion

HER2-positive cancer accounts for 20% of all cases of diagnosed breast cancer. First-line therapy with trastuzumab, pertuzumab and docetaxel has significantly improved overall survival among patients with this type of cancer [39]. However, tumours invariably relapse. Strong evidence demonstrates that the TME plays a crucial role in cancer progression, including breast cancer. Particularly, studies have paid special attention to CAFs as major contributors to the crosstalk between cancer cells and the surrounding stromal cells [15]. CAFs produce and secrete a variety of growth factors and cytokines, with a profound impact on tumoural cell behaviour, thus affecting critical features such as tumour proliferation, metastasis and drug resistance. Many studies suggest that the clinical benefit and overcoming resistance might require targeting CAFs, thus highlighting the relevance of identifying modulable candidate markers in the CAF secretome [14,15,23,40]. Proliferation assays with CM from CAF-200 allowed us to characterise the effect of CAF-200 on the response of two HER2-positive BCCLs to treatment with TPD. Our results suggest that CAF-200 cells secreted a variety of soluble factors with significant impact on the response of BT-474 and EFM-192A cells to therapy. These results are in agreement with those described previously, which reported that CAFs could promote resistance to trastuzumab in HER2-positive BCCLs by activating pathways such us PI3K/AKT/mTOR and JAK/STAT3, as well as by expanding cancer stem cells (CSCs) [21]. However, the effect of CAFs on the response of HER2-positive cancer cells to combined therapy with trastuzumab, pertuzumab and docetaxel has not been described yet. Invasion assays with BT-474 cells demonstrated that soluble factors secreted by CAF-200 also conferred them with a greater invasive capacity, even in presence of drugs. Our results are in agreement with previous reports that demonstrated the influence in vitro of CAFs on BCCL migration capacity, both by CM experiments and by coculturing breast cancer cells with CAFs [41,42,43].

Particularly, CAFs have been described as key players in the promotion of EMT leading to therapy resistance [44,45], as well as drivers of the stem-like properties of CSCs that generate resistance to chemotherapy and targeted therapy [46,47]. Furthermore, the TME may act as a niche for CSCs, regulating self-renewal and differentiation properties [48]. We explored the possible role of the CAF-200 secretome in the promotion of mesenchymal and stem-like phenotypes in BT-474 cells as driver processes of resistance to HER2-targeted therapy. Using spheroids 3-D models, we hypothesised that CAFs could be endowing stem-like properties to tumour cells, therefore increasing resistance to the studied therapies. These results might be linked to our previously described results of proliferation assays. In addition, several EMT-related markers were tested by WB in HER2-positive BT-474 cells. As expected, cells showed an increased expression of mesenchymal markers, such as Snail and fibronectin, when treated with CM[CAF-200/TPD]. These results suggest a potential role of CAF-200 in the promotion of the EMT phenotype in HER2-positive BCCLs—although more markers should be assayed—so exploring its relationship with the response to therapy could reveal potential mechanisms underlying resistance to anti-HER2 therapies.

Moreover, we aimed to explore if the acquisition of resistance to anti-HER2 therapies in BCCLs would occur through changes at the HER2 abundance level. The addition of CM[CAF-200/TPD] reversed the decrease produced by anti-HER2 therapy, as well as restored phosphorylation of HER2 to basal levels, at least partially. These data agree with our previous work [34], where the decrease in the HER2 phosphorylation level was lower in those cells with trastuzumab-acquired resistance. In addition, the phosphorylation of AKT (Ser473 and Thr308) slightly increased when cells were cultured with CM[CAF-200/TPD], suggesting activation of the PI3K/AKT/mTOR pathway, which has been reported to be associated with resistance to HER2-targeted therapies [35,49,50]. Interestingly, CM[CAF-200/TPD] significantly increased the expression and phosphorylation levels of STAT3 in BT-474 cells through the upregulation of cytokines. The STAT3 transcription factor has been extensively studied as a transcriptional regulator in many diseases, including cancers. The phosphorylation of STAT3 is an intracellular mediator of cytokine signalling [51]. STAT3 activation has been previously associated with trastuzumab and trastuzumab–emtansine resistance in HER2-positive breast cancer [28,52]. Specifically, several studies have recently described the role of STAT3 from the TME cells in regulating the immune response (as reviewed in [53]). Thus, signals from CAFs (cytokines, such as IL-6, VEGF and LIF; as well as chemokines, such as CCL2 and CXCL12 and also growth factors, such as EGF, FGF and IGF), recruit myeloid cells to the TME while inducing, via STAT3, an immunosuppressive phenotype, ultimately causing a disruption of the immune response (reviewed in [54]). These descriptions fit with our findings, as we observed that some of the cytokines secreted by the CAF-200s and selected in our analyses were precisely IL-6, LIF and CCL2. In our cellular model of tumour cell–fibroblast interaction, there is obviously no consequence for the regulation of the immune response, but we hypothesise that the activation of these pathways, as evidenced by the overexpression of IL-6, LIF, CCL2 and others and the subsequent activation by phosphorylation of STAT3 in BT-474, could be relevant in the acquisition of resistance to anti-HER2 therapy in the tumour cell. Interestingly, recent studies suggest that aberrant activation of the STAT3 pathway induced by novel activators may play a key role in drug resistance and recurrence [55]. This is the case for some of the proteins (not necessarily cytokines, but tyrosine kinases, enzymes, chaperones and others) selected in our analysis: SERPINE1, for example, has been described as a mediator of EMT and a metastasis through STAT3 signalling [56]; ADAM12, as a regulator of a proangiogenic TME [57]; and chaperones such as HSP27, HSP70, HSP90 and HSP110 control the activation of STAT3/5 to stimulate cancer cell proliferation and survival, immunosuppression and eventually tumour progression [58].

Broadly speaking, the *modus operandi* of CAFs promotes tumourigenic features in the TME in two ways: either by directly initiating the remodelling of the extracellular matrix or by secreting cytokines that exert a paracrine effect on cancer cell behaviour. An integrated multiomics analysis of secretome from the CAF-200 cell line allowed us to characterise soluble proteins, cytokines and growth factors, as well as miRNAs with a potential role in HER2-targeted therapy resistance. Our strategy revealed significantly modulated proteins and cytokines belonging to relevant pathways associated with oncogenic processes involved in increased cell proliferation, cell growth and cell survival. We found some overlap between the techniques used, as several of the proteins found in our label-free analysis were cytokines and growth factors previously identified by antibody arrays (e.g., CCL2, IL-6 and IGFBP4); this correlation of results confirmed the robustness of the multiomics approach.

One of the basic intercellular communication procedures that regulate signalling flux in the TME is the exchange of small RNA-derived molecules. Specifically, CAF-derived microRNAs modulate tumour cell growth, proliferation, invasion, migration and chemoresistance. Our analysis revealed few miRNAs involved in the regulation of the TME response, although several of them have been described in the literature in relation to drug resistance in cancer. miR-199b-5p is the most appealing candidate, as it has been proposed as a therapeutic target in HER2-positive breast cancer [29]. In breast cancer patients, it has been shown to be deregulated compared to the corresponding adjacent normal tissues and was also correlated with poor prognosis [59]. miR-130a-3p, on the other hand, has been described to act as an oncogene, promoting tumourigenesis by targeting tumour suppressor genes such as *RUNX3* and *PTEN* [60]. Interestingly, miR-130a-3p was previously described in TNBC as a significantly overexpressed miRNA related to the promotion of EMT processes [61] and the resistance to various drugs, such as gemcitabine [62] and cisplatin [63]. The other candidates in our analysis are downregulated miRNAs, some of which have already been described as modulators of tumour progression in breast cancer. Some of them, such as miR-4281 and miR-23b-3p, have been described as playing apparently contradictory roles. In some cases, they have been associated with a protumourigenic role in breast cancer when they are upregulated, enhancing HER2 expression and tamoxifen resistance [64]. Conversely, in other instances, the downregulation of miR-23b-3p has been proposed as a possible mechanism of trastuzumab resistance in HER2-positive breast cancer cells [65].

Some of the secretome components that we found in our parallel antibody array/mass spectrometry approach have already been widely explored in breast tumours, among others. This is the case of IL-6, which has been shown to have a direct effect on cancer cell growth and survival, as well as on resistance to therapy [66]. Additionally, IL-6 is also well described as a regulator of inflammatory and immune responses in cancer [67]. Importantly, IL-6 activates STAT3—as we found in our experiments—giving the IL-6/JAK/STAT3 pathway a key role in tumour progression in a variety of types, including breast cancer [68,69]. Other interleukins activated in our model have also been described in the literature as promoting tumourigenesis-associated processes, such as the leukaemia inhibitory factor (LIF). It can activate multiple pathways, including the JAK/STAT3 and the AKT/mTOR pathways, and is frequently overexpressed in breast cancer and other tumour types, playing a role in promoting tumour progression, metastasis and EMT phenotype [70,71]. Additionally, a wealth of evidence has identified in breast tumours a subset of CAFs characterised by a high expression and secretion of LIF, stimulating a TME with a proinvasive phenotype [72]. Interestingly, a functional annotation of the results of our multiomics approach revealed a correlation of some of the components of the CAF secretome with immune-related pathways, even though our cellular model lacked immune cells. One of those is the chemokine C-C ligand 2 (CCL2), which has been shown to play a key role in both cancer and stromal cells. In tumour cells, it promotes cell proliferation and invasion, as well as inflammation and angiogenesis [73]. CCL2 production by CAFs, which is often upregulated, has been shown to induce angiogenesis and is associated with the promotion of CSC characteristics in breast cancer [74]. Furthermore, CAFs may also regulate monocyte and macrophage recruitment and differentiation via secretion of CCL2, contributing to immune suppression and metastasis in breast cancer cells [75]. The fibroblast growth factor 7 (FGF-7), on the other hand, has been described in the stromal TME with paracrine effect on epithelial cells, specifically on cell proliferation properties [76]. Importantly, recent work on breast tumours showed in vivo that reducing the production and secretion of FGF-7 by CAFs, together with other factors, could diminish cell growth and cancer progression [77]. The coexpression of FGFs and their receptors correlates significantly with a poor prognosis of patients, so FGFR-specific inhibitors have been developed in recent years [78].

Since the purpose of our research was to uncover possible causal mechanisms of resistance to TPD combination therapy, we decided to use a bioinformatic analysis to investigate the potential relationship with resistance of some of the components of the CAF-200 secretome, in particular, proteins associated with the immune response profile. The *PA2G4* gene, for instance, which codes for the ErbB signal transduction protein (EBP1), has been linked to the resistance to hormone and tyrosine kinase directed therapies [79]. Its role as a regulator of ErbB3 makes EBP1 a contributor to breast cancer progression and treatment resistance, as it controls, for example, the ErbB2 protein levels and tamoxifen sensitivity in breast cancer cells. Several of the other proteins identified in our study belong to the heat shock protein family (HSP90-alpha, HSP90-beta and DnaJ homolog subfamily C member 3). In addition to their role as chaperones under conditions of cellular stress or development, and as upstream regulators of many oncogenes, these proteins have also been described in the literature in relation to drug resistance in cancer [80]. Strategies to inhibit their expression restore docetaxel sensitivity. In breast cancer, for instance, HSP90 is thought to simultaneously control the immune surveillance of natural killer cells and the persistence of drug-treated tumour cells [81]. TXN, on the other hand, has been associated in breast cancer with resistance to both docetaxel and trastuzumab [82]. A study with breast cancer patients treated with docetaxel revealed that the TXN expression significantly increased after docetaxel therapy, although there was no significant association between the extent of increase in the TXN expression and response [36]. In another study, high levels of reduced TXN caused the failure of trastuzumab to activate phosphatase and tensin homolog (PTEN), and TXN inhibition was required for sensitization of breast cancer cells resistant to anti-HER2 therapy. Other genes/proteins in our selection have also been described in relation to resistance. High mobility group box 1 (HMGB1) has been shown to promote autophagy protection in response to docetaxel therapy in lung cancer through the activation of the ERK signalling pathway [37]. Recently, S100A family proteins have been shown to be associated with drug resistance in breast cancer [83]. Specifically, the activation of p53 causes differential regulation of S100A proteins, resulting in changes in calcium regulation in favour of prosurvival functions. CCL2, already mentioned, may play a role in the docetaxel resistance in cancer through the activation of the PI3K/AKT pathway and inhibition of apoptosis [84]. We acknowledge the limitations of this analysis: It is only a mathematical study that does not add experimental information. We could not obtain pertuzumab sensitivity/resistance data. The analysis of each drug was performed on an individual basis (and not in combination as TPD, as is the aim of our work). It was performed on a molecular profile not specifically linked to the resistance mechanisms—especially to trastuzumab—described and well accepted for years. Finally, it was performed with data from BCCLs and not CAFs. However, we believe that this analysis may be of some use in shedding light on the involvement of certain genes in the mechanisms of drug resistance in cancer. In any case, these results should be taken with caution, thus a certain distance between these scenarios and the clinical reality of patients is to be expected.

Additionally, we have revealed the association of some of the gene/protein clusters selected in our analysis with RFS survival in HER2-positive breast cancer. Some of the data show promising results, and it might be interesting to combine these values with predictors commonly used in clinical practice to see if they add prognostic and predictive information.

Finally, another essential aspect to consider in the study of CAFs is that, as major components of the tumour stroma, these cells are subject to the same pharmacological treatment as cancer cells. Therefore, the study of their response to antitumour therapy has a dual interest: First, to discover whether treatment modulates the release of secretions into the extracellular milieu, thus conditioning the exchange of signals with cancer cells and, therefore, tumour progression and Second, to study whether treatment alters the physiology of CAFs in the context of TME, especially immunosuppression and immunosurveillance [85]. In both scenarios, it is therefore essential to decipher the molecular profile of the secretome in order to understand the potential alterations that therapy is able to induce in the identity, abundance and patterns of nongenetic modifications in the molecules released to the ECM by CAFs. Numerous drugs have been tested in in vitro, translational and clinical trials, some specific to CAFs and their communication with the tumour cell (Class 2 agents) and others that also affect other cell types (Classes 1 and 3) [86]. Furthermore, in recent years, several approaches have been developed to combine such specific antiCAF treatments with antitumour immunotherapies [54]. Despite the promising results of the first preclinical trials of combining checkpoint blockade immunotherapies with specific antiCAF treatments, many questions remain to be answered (about the heterogeneity of CAFs, the role of each subtype in immunosuppression, their plasticity in drug response, the regulation of immunosurveillance activity of CAFs in response to treatments and the translation of many of the in vitro results to patients) before integrating targeted antiCAF therapies into clinical practice.

The present study has a fundamentally exploratory and descriptive aim about the role that certain CAF secretome molecules may play in the generation of resistance to anti-HER2-targeted therapy in breast cancer. Some of the candidates presented in this article are currently being validated in our laboratory by functional studies to elucidate the most relevant mechanisms of action involved in this resistance. In fact, we are trying to overcome one of the limitations of our study, which is based on the results generated in a single CAF line: we currently have more than 10 fibroblast lines obtained from HER2-positive breast tumour samples and several BCCLs with HER2-positive phenotype, where we intend to confirm the preclinical utility of the results presented here.

## 4. Materials and Methods

### 4.1. Cell Cultures and Treatments

Human breast cancer cell line BT-474 (HTB-20) was purchased from the American Type Culture Collection, and EFM-192A (ACC-258) was obtained from the German Tissue repository DSMZ. The cell lines were checked for authentication every 6 months, either by using the Cell Line Authentication service at LGC Standards (London, UK) (tracking no: 710259498; 710272355) or by running a home-made mutational profiling assay [34]. BT-474 cells were maintained in DMEM/F-12 (Sigma Aldrich, Steinheim, Germany) supplemented with 10% heat-inactivated foetal bovine serum (Gibco, Thermo Fisher Scientific, Waltham, MA, USA), 2 mmol/L glutamine (GlutaMAX, Gibco) and 1% penicillin G-streptomycin (P/S, Gibco). EFM-192A cells were cultured in RPMI 1640 (Gibco) with 20% heat-inactivated FBS and 1% P/S. All cell lines were grown as monolayers at 37 °C under humidified atmosphere with 5% CO_2_ and were tested for mycoplasma contamination using the previously described protocol [34]. The human cancer-associated fibroblast cell line CAF-200 was obtained from a tumourectomy performed in a HER2-positive patient and immortalised using a retroviral vector expressing hTERT, as previously reported [22]. The CAF-200 cell line was cultured in DMEM-high glucose (Sigma Aldrich) supplemented with 10% FBS, 2 mmol/L glutamine and 1% P/S.

Recombinant humanised monoclonal HER2 antibody trastuzumab (a concentration of 15 μg/mL was selected as indicated elsewhere [34]) was supplied by the pharmacy of our hospital; pertuzumab (a concentration of 20 μg/mL was used in accordance with reports in the literature) was obtained from Genentech (San Francisco, CA, USA) and docetaxel (employed at a dose of 0.5 nM) was obtained from Selleckchem (Selleckchem Spain, Madrid, Spain).

### 4.2. Generation of Conditioned Medium (CM) Samples

In order to obtain CM, CAF-200 cells were seeded in T-175 flasks until they reached 50–60% confluence. Then, cells were washed with PBS and subsequently incubated with a fresh medium plus (except control flasks) a combined treatment (TPD) of trastuzumab (15 μg/mL), pertuzumab (20 μg/mL) and docetaxel (0.5 nM) for 72 h. Then, for cytokines arrays, proteomic and miRNA analyses, the control (CM[CAF-200]) and treated (CM[CAF-200/TPD]) flasks were washed with PBS and afterwards the cells were cultured in a serum-free medium for 24 h. Finally, CM was collected at this point and centrifuged at 2000 rpm for 2 min to discard cell debris; then, CM was filtered through a 0.22 μM filter (Millex^®^-GP, Merck Millipore Ltd. Tullagreen, Carrigtwohill, Co. Cork, Ireland). For proliferation and migration assays, tumour spheroid formation and blottings, we used a CM[CAF-200/TPD] without serum starvation.

### 4.3. Cell Proliferation Assays

To determine cell proliferation rates of the breast cancer cell lines, tumour cells were seeded in triplicate in a 12-well plate at a density of 50,000 cells per well and allowed to adhere for 24 h in a complete medium. Then, cells were grown for 7 days in either a fresh medium or in a mixture of CM[CAF-200/TPD] with a fresh medium (2:1) and treated (or not) with TPD. The appropriate culture medium and treatments were replaced every 3 days (except docetaxel, which was only maintained for the first 72 h).

### 4.4. Tumour Spheroid Formation Assay

The in vitro spheroid formation assays were performed using 6-well ultra-low attachment plates (Corning, Kennebunk, ME, USA). BT-474 cells were seeded at a density of 2500 cells per well in 2 mL of the corresponding culture medium (either DMEM/F-12 or CM[CAF-200/TPD] mixed with a fresh medium), without or with TPD treatment. Cells were resuspended with a p1000 micropipette and then cultured for 7 days. On the last day of culture, each well was divided into quadrants to facilitate the counting of spheroids. Each well was counted 3 times, and three independent experiments were performed.

### 4.5. Transwell Migration Assays

Migration assays were performed using 24-well plates with transwell permeable supports of a 6.5 mm insert and a polycarbonate membrane with an 8 μm pore size (Corning, Corning, NY, USA). After serum starvation for 24 h, BT-474 cells were seeded in the upper chamber at a density of 2000 cells per insert, in 0.15 mL of DMEM/F-12. A volume of 0.5 mL of the corresponding medium (DMEM/F-12 or CM[CAF-200/TPD], mixed with fresh medium), without or with TPD treatment, was placed in the bottom well. After incubation at 37 °C in a 5% CO_2_ atmosphere for 24 h, the migrated cells on the lower surface were stained using crystal violet and counted under a light microscope. Ten randomised fields (magnification 20×) were counted. Three independent experiments were performed.

### 4.6. Protein Extraction and Quantification

Cells were seeded in 6-well plates at a density of 500,000 cells/well, in the presence of the corresponding medium (DMEM/F-12 or CM[CAF-200/TPD] for 72 h. Then, cells were washed with 3 mL PBS at RT. Next, cells were scraped in the presence of a 150 μL lysis buffer (RIPA buffer plus peptidase and phosphatase inhibitors) at 4 °C and transferred to a 1.5 mL tube. Cells were incubated in the lysis buffer for 10 min at 4 °C and sonicated afterwards. Then, the cell lysate was spun at 13,000× *g* for 10 min at 4 °C, and the supernatant was retained and stored. Protein extracts were quantified using the Pierce BCA protein assay kit (Thermo Fisher Scientific) following manufacturer’s instructions.

### 4.7. Western Blotting (WB)

Protein aliquots were prepared at 1 μg/μL in a 4× Laemmli loading buffer and boiled at 95 °C for 5 min. Twenty μL of protein extract was loaded in a 10% polyacrylamide gel (SDS-PAGE). Next, proteins were transferred to a nitrocellulose membrane for 90 min at 130 V and 4 °C. The membrane was blocked (5% milk in PBST 1×) for 1 h and then incubated with the primary antibody at RT o/n under agitation at 4 °C. The antibodies employed were as follows: HER2, p-HER2 Tyr1221/1222, p44/42 MAPK (ERK1/2), p-p44/42 MAPK (ERK1/2) Thr202/Tyr204, AKT, p-AKT Thr308, p-AKT Ser473, STAT3, p-STAT3 Tyr705, SNAIL, E-cadherin (Cell Signaling, Danvers, MA, USA), occludin, β-actin (Sigma Aldrich) and fibronectin (Abcam, Cambridge, UK). All antibodies were used at 1:1000 and were rabbit monoclonal antibodies, except β-actin, which was used at 1:5000 and was a mouse monoclonal antibody. All antibodies were prepared in 5% milk in PBS 1×. Then the membranes were washed 4 × 5 min in PBST and incubated with a secondary antibody (diluted in 5% milk in PBST 1×) at RT for 1 h. The ECL antimouse and ECL antirabbit secondary antibodies attached to peroxidase (HRP; GE Healthcare, Chicago, IL, USA) were used at a concentration of 1:5000. The membranes were washed again for 4 × 5 min and submerged in the detection reagent (Immobilon Crescendo Western HRP substrate, Merck Millipore) for 2 min prior to developing on a photographic film. The densitometry and quantification of proteins were measured using the ImageJ software. The original WB images can be found as Supplementary Material (Figure S5).

### 4.8. RNA Isolation

Isolation of exosomal RNA (including miRNAs) from CM was carried out using the exoRNeasy Serum/Plasma Midi Kit (Qiagen GmbH, Hilden, Germany), according to manufacturer’s instructions. RNA purity and integrity were assessed using a NanoDrop 2000 (NanoDrop Technologies, Wilmington, DE, USA), as well as the Agilent 2100 Bioanalyzer (Agilent Technologies, Santa Clara, CA, USA).

### 4.9. RNA Library Preparation and Sequencing

RNA libraries were prepared starting from 150 ng of total RNA using the SMARTer^®^ smRNA-Seq Kit for Illumina^®^ protocol (Clontech Laboratories Inc., San Jose, CA, USA) according to manufacturer’s instructions. Library validation was performed by the Agilent 2100 Bioanalyzer, and enrichment of smRNA (inserts < 150 bp) was carried out by size selection using Agencourt AMPure XP Beads. Library sequencing was performed on an Illumina MiSeq v3 flow cell (2 × 75 bp) (Illumina Inc., San Diego, CA, USA). All procedures were performed at the Genomics Core Facility of the Universitat Pompeu Fabra (Spain).

### 4.10. MiRNA Identification and Differential Expression Analysis

Raw sequencing reads were mapped with STAR v 2.6.0a [87] onto the miRBase v21. The table of counts was obtained with the FeatureCounts function in the package subread, version 1.5.1 [88]. Differential expression analysis was assessed using the voom transformation on the TMM factors. A limma moderated t-statistics model was applied using an R package version 3.30.13 and R version 3.5.2 [89]. Correction for multiple comparisons was performed using a false discovery rate (FDR) by adjusting the *p*-value [90]. The identification and analysis of differentially expressed miRNAs were carried out at the Servei d’Anàlisi de Microarrays of MARGenomics (Spain).

### 4.11. MiRNA-199b Transfection and Cell Proliferation Assay

For transfection experiments, BT-474 cells were seeded in 6-well plates and cultured in a fresh medium without antibiotics. Cells were transfected at a confluence of 50–60% with 10 μL of Lipofectamine 2000 (Life Technologies, Thermo Fisher Scientific) and 20 nM of an miR-199b-specific mirVanaTM miRNA Mimic (Ambion, Austin, TX, USA) and treated without or with TPD. After 72 h, cells were washed with PBS, the fresh medium was replaced, and the transfection protocol was repeated as described (treatment with 15 μg/mL trastuzumab and 20 μg/mL pertuzumab was also replaced). After 48 h, cells were then harvested by trypsinisation and counted using the TC20 Automated Cell Counter (BioRad, Hercules, CA, USA).

### 4.12. Cytokine Arrays

CM[CAF-200] and CM[CAF-200/TPD] samples were prepared. Samples were incubated with the membrane-based antibody array Human Cytokine Array C5 (RayBiotech Life Inc., Peachtree Corners, GA, USA) at 4 °C o/n following manufacturer’s instructions. The expression levels of cytokines were determined, and signal intensities were analysed using the ImageJ software programme. Several calculations were performed to estimate the differences between treated and untreated samples: relative intensity, Student’s *t*-test, differential intensity, relative fold change, fold change and number of hits, understood as the number of parameters (among the preceding ones) for which a given cytokine was found to be significantly different between the control and treated samples. Two independent experiments were performed.

### 4.13. Gene Ontology (GO) Analysis

We performed functional profiling of the proteomic data using the Gene Ontology resource from the GO Consortium server [91]. Functional enrichment analysis of overrepresented ontology terms was performed with the GO Enrichment Analysis tool powered by PANTHER [92]. It allowed us to categorise the molecular function, biological process and cellular localization of the unique proteins identified in this study. Only those terms showing FDR < 0.05 were considered statistically significant.

### 4.14. Mass Spectrometry Analysis

Fresh CM samples were concentrated from 20 mL to approximately 2 mL by using Vivaspin^®^ 20 Centrifugal Concentrators 10K (Sartorius Stedim Lab Ltd., Stonehouse, UK). Proteinase K 500 μg/mL in PBS was added to break the exosomal membranes. A total of 6 samples corresponding to three biological replicates of each group (control and treated samples) were analysed. The proteomic analysis was performed at the Proteomics Unit of the Complutense University of Madrid (Spain) as reported elsewhere [93]. Briefly, 50 μg of each protein extract was concentrated in a stacking gel. The bands of proteins were cut from the gel, reduced, alkylated and trypsin digested o/n. Then the peptides from the digested proteins were desalted and concentrated with C18 reverse phase chromatography, eluted, freeze-dried in speed-vac and resuspended in acetonitrile/formic acid. The desalted peptides were analysed by a reverse phase liquid chromatography electrospray ionisation tandem mass spectrometry (RP-LC-ESI-MS/MS) in an EASY-nLC 1000 System coupled to the Q-Exactive HF mass spectrometer (an ultra-high-field mass orbitrap analyser) through the Nano-Easy spray source (all from Thermo Scientific, Bremen, Germany). All data were acquired using data-dependent acquisition (DDA) and in positive mode with the Xcalibur 4.0 software (Thermo Fisher Scientific).

### 4.15. Protein Identification and Quantification

Peptide identifications from raw data were carried out using the Mascot v. 2.6.1 (MatrixScience, London, UK) search engine through the Protein Discoverer 2.2 Software (Thermo Fisher Scientific). A database search was performed against the UniProtKB/Swiss-Prot human database release 2019_05 (https://www.uniprot.org, accessed on 1 June 2019). To determine the abundance of the peptides and proteins identified in different isolates, a label-free experiment based on precursor signal intensity was performed. After the analyses were completed, a final report presented the list of peptide groups and proteins with normalised abundances and selected ratios.

### 4.16. Protein Data Analysis

The Proteome Discoverer application includes a statistical feature (Anova Background) for assessing the significance of differential expression by providing *p*-values and adjusted *p*-values (q-values) for those ratios. Only proteins identified with high confidence (FDR < 1%) with at least one unique peptide, abundance ratio variability < 30%, q-value < 0.05 and fold change > 1.5 were considered to be differentially expressed between groups. The mass spectrometry proteomics data have been deposited in the ProteomeXchange Consortium via the PRIDE partner repository with the dataset identifier PXD025556.

Proteome Discoverer includes a principal component analysis (PCA) to identify the major components in a protein dataset using abundance normalised values. The input data used for PCA were the master proteins identified with high confidence in the database, without taking contaminating proteins into account.

### 4.17. Gene Set Enrichment Analysis

Gene Set Enrichment Analysis (GSEA) was performed [94]. Functional enrichment was applied using annotations from the MsigDB, Reactome, KEGG and NCI databases. Genes were ranked based on the limma moderated t-statistic [89]. After Kolmogorov–Smirnoff testing, those gene sets showing FDR < 0.05 were considered enriched between classes under comparison.

### 4.18. Kaplan-Meier Plotter Analysis

We estimated the prognostic value of different protein clusters in HER2-positive breast cancer using an online database, Kaplan–Meier Plotter (www.kmplot.com, accessed on May 2021) [95], which contained gene expression data and survival information of more than 15,000 cancer patients. Gene expression data and relapse free (RFS) and overall survival (OS) information are publicly available from the Gene Expression Omnibus (GEO, http://www.ncbi.nlm.nih.gov/geo/, accessed on 1 May 2021) and The Cancer Genome Atlas (TCGA). To calculate the survival estimate of a group of candidate markers, patient samples were split into two groups by median expression (high versus low expression) and assessed by a Kaplan–Meier survival plot, with the hazard ratio (HR) with 95% confidence intervals and log rank *p*-value.

### 4.19. Analysis of Drug Sensitivity and Resistance

To investigate the effect of our gene signature on drug sensitivity in a panel of breast cancer cell lines, we integrated data from the Cancer Cell Line Encyclopedia (CCLE) [96], the Genomic markers screening of Drug Sensitivity in Cancer (GDSC) [33] drug response signature and our own sensitivity data [34]. CCLE expression values for these genes were compared with either GDSC IC50 values for docetaxel or the cellular growth rate for trastuzumab in HER2-positive breast cancer cell lines (19 parental cell lines plus 4 derived resistant lines) by linear regression with ANOVA < 0.01.

### 4.20. Statistical Analysis

All measured data are expressed as means ± standard deviations of at least three replicates (unless otherwise indicated). Statistical significance was analysed by a two-tailed Student’s *t*-test (*: *p*-value < 0.05, **: *p*-value < 0.01, ***: *p*-value < 0.001). This work was performed in accordance with the Reporting Recommendations for Tumour Marker Prognostic Studies (REMARK) guidelines [97].

## 5. Conclusions

In summary, our study reveals the impact of CAFs on the regulation of HER2-positive breast cancer cells, demonstrating in vitro their ability to promote resistance to HER2-targeted therapies. Our results demonstrate that CAFs exert an essential contribution via paracrine signalling in resistance to trastuzumab- and pertuzumab-based anti-HER2 therapy, even in the presence of taxane chemotherapy. Our data also demonstrate in vitro the positive effect of the CAFs secretome in promoting several therapy resistance-related features, such as invasiveness, tumour spheroid formation or mesenchymal phenotype. We carried out a multiomics strategy to characterise the secretome of CAFs under treatment with an anti-HER2 therapy combined with taxane-based chemotherapy. The combination of miRNA analysis, cytokine arrays and label-free LC-MS/MS quantification allowed us to identify candidates with a potential role in the generation of resistance in HER2-positive breast cancer. The integration of all these data allowed us to establish a series of molecular signatures based on the functional groupings of proteins. These protein clusters revealed, on the one hand, a correlation with RFS data for breast cancer patients, and, on the other, we observed interesting links between our clusters and a number of proteins implicated in trastuzumab and docetaxel sensitivity. All these data emphasise the potential utility of these candidates secreted by CAF, as they could emerge as therapeutic targets for the modulation of the resistant phenotype, supporting the improvement of the clinical scenario in HER2-positive patients.

## Figures and Tables

**Table 1:** Therapy with TPD induced changes in secreted miRNA pattern of CAF-200. Up- and downregulated miRNAs with at least 1.5-fold found in CAF-200 secretome after treatment with triple TPD therapy.

miRNA	logFC	*p*-Value
hsa-mir-130a-3p	1.96	0.03
hsa-let-7b-3p	1.79	0.04
hsa-mir-199b-5p	1.76	0.03
hsa-mir-4787-3p	−2.19	0.02
hsa-mir-4281	−2.04	0.04
hsa-mir-4800-3p	−1.97	0.03
hsa-mir-23b-3p	−1.95	0.02
hsa-mir-4485-5p	−1.89	0.04
hsa-mir-7854-3p	−1.79	0.03

**Table 2:** List of cytokines/chemokines present and quantified (by Image J) in the membrane antibody array (Human Cytokine Antibody Array 5) stained with CM. The intensity column shows the average of the values of the treated samples. The other parameters provide different measures of the differences between treated and control samples. # hits: number of parameters for which a given cytokine was found to be significantly different between control and treated samples.

Cytokine	Intensity	*p*-Value	Differential Intensity	Relative Fold Change	log2 FC	# Hits
Angiogenin	0.3199	0.0028	−0.1331	−0.2938	−0.5019	5
BDNF	0.4531	0.4293	−0.0297	−0.0616	−0.0917	1
BLC (CXCL13)	0.0715	0.1307	−0.0895	−0.5558	−1.1708	0
Ck beta 8-1 (CCL23)	0.0322	0.1344	−0.1055	−0.7660	−2.0955	0
EGF	0.0100	0.0904	−0.1008	−0.9097	−3.4698	0
ENA-78 (CXCL5)	0.0464	0.1580	−0.0459	−0.4973	−0.9922	0
Eotaxin-1 (CCL11)	0.0208	0.1268	−0.0918	−0.8155	−2.4387	0
Eotaxin-2 (CCL24)	0.0100	0.1782	−0.0778	−0.8861	−3.1341	0
Eotaxin-3 (CCL26)	0.0100	0.0456	−0.0325	−0.7647	−2.0872	2
FGF-4	0.1738	0.4402	0.0288	0.1987	0.2614	0
FGF-6	0.1882	0.0561	0.0600	0.4679	0.5538	1
FGF-7 (KGF)	0.0997	0.1638	0.0436	0.7776	0.8300	4
FGF-9	0.2474	0.5959	0.0315	0.1460	0.1966	1
FLT-3 Ligand	0.1239	0.5896	−0.0123	−0.0906	−0.1370	0
Fractalkine (CX3CL1)	0.1396	0.1526	−0.0589	−0.2969	−0.5082	0
G-CSF	0.0100	0.1813	−0.0391	−0.7962	−2.2948	0
GDNF	0.0393	0.0590	−0.1486	−0.7907	−2.2565	3
GM-CSF	0.0100	0.0739	−0.0320	−0.7621	−2.0716	0
GPC-2 (CXCL6)	0.0466	0.0889	−0.1052	−0.6931	−1.7041	0
GRO a/b/g	0.1577	0.0313	−0.1014	−0.3913	−0.7162	2
GRO alpha (CXCL1)	0.0100	0.1253	−0.0267	−0.7279	−1.8775	0
HGF	0.0100	0.1463	−0.0363	−0.7842	−2.2124	0
I-309 (CCL1)	0.0338	0.5121	−0.0289	−0.4605	−0.8904	0
IFN-gamma	0.1881	0.9113	0.0051	0.0281	0.0400	0
IGF-1	0.0100	0.0384	−0.0750	−0.8824	−3.0874	3
IGFBP-1	0.0100	0.2575	−0.0365	−0.7851	−2.2180	0
IGFBP-2	0.0253	0.0948	−0.0776	−0.7542	−2.0247	0
IGFBP-3	0.1122	0.9345	0.0010	0.0092	0.0133	0
IGFBP-4	0.1027	0.2169	−0.0222	−0.1774	−0.2817	0
IL-1 alpha (IL-1 F1)	0.0770	0.8593	−0.0083	−0.0977	−0.1483	0
IL-1 beta (IL-1 F2)	0.2268	0.3119	0.0318	0.1633	0.2182	0
IL-10	0.0100	NA	0.0000	0.0000	0.0000	0
IL-12 (p40/p70)	0.1481	0.0983	−0.0269	−0.1536	−0.2406	0
IL-13	0.0654	0.3267	0.0323	0.9763	0.9828	1
IL-15	0.2774	0.2844	0.0478	0.2081	0.2727	2
IL-16	0.1368	0.2513	−0.0505	−0.2697	−0.4535	0
IL-2	0.1239	0.5033	0.0271	0.2799	0.3561	0
IL-3	0.1964	0.6411	0.0224	0.1290	0.1751	0
IL-4	0.0869	0.2814	−0.0215	−0.1984	−0.3191	0
IL-5	0.0100	0.1043	−0.0978	−0.9073	−3.4306	3
IL-6	1.8106	0.5854	−0.0546	−0.0293	−0.0429	1
IL-7	0.0100	0.0949	−0.1245	−0.9256	−3.7491	4
IL-8 (CXCL8)	1.0915	0.9680	0.0061	0.0057	0.0082	1
IP-10 (CXCL10)	0.6526	0.2375	0.0728	0.1256	0.1707	4
Leptin	0.1577	0.2038	−0.0463	−0.2268	−0.3711	0
LIF	0.3174	0.5290	0.0531	0.2011	0.2643	4
LIGHT (TNFSF14)	0.1716	0.7360	−0.0059	−0.0331	−0.0486	0
MCP-1 (CCL2)	1.5040	0.1989	0.1576	0.1170	0.1596	4
MCP-2 (CCL8)	0.0707	0.0456	−0.0596	−0.4575	−0.8824	1
MCP-3 (CCL7)	0.0100	0.0017	−0.0681	−0.8719	−2.9649	2
MCP-4 (CCL13)	0.0676	0.1183	−0.0606	−0.4727	−0.9232	0
M-CSF	0.0577	0.0341	−0.1840	−0.7613	−2.0669	2
MDC (CCL22)	0.0100	0.0452	−0.0986	−0.9079	−3.4404	1
MIF	0.0362	0.0042	−0.1472	−0.8025	−2.3398	5
MIG (CXCL9)	0.0100	0.1174	−0.0204	−0.6708	−1.6028	0
MIP-1 beta (CCL4)	0.2621	0.0575	−0.0705	−0.2120	−0.3437	1
MIP-1 delta	0.0100	0.0006	−0.0684	−0.8725	−2.9711	3
MIP-3 alpha (CCL20)	0.0100	0.0146	−0.0516	−0.8377	−2.6232	1
NAP-2 (CXCL7)	0.0100	0.0856	−0.0692	−0.8737	−2.9850	0
NT-3	0.3264	0.3826	−0.0320	−0.0894	−0.1351	1
NT-4	0.0807	0.4584	−0.0204	−0.2020	−0.3256	0
OPG (TNFR SF 11)	1.3666	0.6382	0.0410	0.0309	0.0439	1
OPN (SSP1)	0.3109	0.1552	−0.0895	−0.2235	−0.3649	1
OSM	0.4078	0.6803	0.0178	0.0455	0.0642	1
PARC	0.1914	0.8600	−0.0119	−0.0586	−0.0871	0
PDGF-BB	0.0948	0.3120	−0.0265	−0.2187	−0.3561	0
PLGF	0.1465	0.3941	0.0627	0.7481	0.8058	0
RANTES (CCL5)	0.2646	0.6313	0.0288	0.1221	0.1662	1
SCF	0.1688	0.2936	0.0226	0.1546	0.2073	1
SDF-1 alpha (CXCL12)	0.2151	0.9245	−0.0024	−0.0112	−0.0163	0
TARC (CCL17)	0.3281	0.5687	−0.0430	−0.1158	−0.1775	1
TGF beta 1	0.0544	0.0192	−0.0718	−0.5692	−1.2149	1
TGF beta 2	0.6476	0.0122	0.1226	0.2335	0.3028	5
TGF beta 3	0.1224	0.3794	−0.0171	−0.1228	−0.1890	0
TIMP-1	0.6717	0.4984	−0.0302	−0.0430	−0.0634	1
TIMP-2	1.3389	0.1198	−0.0474	−0.0342	−0.0502	1
TNF alpha	0.0100	0.0013	−0.0729	−0.8794	−3.0519	3
TNF beta (TNF SF 1B)	0.0536	0.0996	−0.0943	−0.6377	−1.4649	0
TPO	0.1176	0.0026	0.0337	0.4011	0.4865	4
VEGF-A	0.2579	0.6450	0.0224	0.0952	0.1312	1

**Table 3:** List of pathways found in the Reactome analysis, with indication of the selected CAF-200 secretome proteins that were associated with each pathway. For reasons of space, only the first 20 pathways are listed.

Pathway Name	Pathway Identifier	Proteins from Study Found in Pathway	no. Proteins in Study (Total)
Immune System	R-HSA-168256	AP1B1, CCL2, CTSA, DNAJC3, HMGB1, HSP90AA1, HSP90AB1, PA2G4, S100A11, TXN, YWHAZ	11 (2895)
Innate Immune System	R-HSA-168249	CTSA, DNAJC3, HMGB1, HSP90AA1, HSP90AB1, PA2G4, S100A11, TXN	8 (1331)
Neutrophil degranulation	R-HSA-6798695	CTSA, DNAJC3, HMGB1, HSP90AA1, HSP90AB1, PA2G4, S100A11	7 (480)
Disease	R-HSA-1643685	AP1B1, APOA1, CTSA, DNAJC3, HSP90AA1, HSP90AB1, TXN	7 (2512)
Signal Transduction	R-HSA-162582	APOA1, CCL2, HSP90AA1, HSP90AB1, SERPINE1, YWHAH, YWHAZ	7 (3421)
Metabolism of proteins	R-HSA-392499	APOA1, CCL2, CTSA, DNAJC3, EIF3F, TXN	6 (2355)
Vesicle-mediated transport	R-HSA-5653656	AP1B1, APOA1, HSP90AA1, YWHAH, YWHAZ	5 (825)
Infectious disease	R-HSA-5663205	AP1B1, DNAJC3, HSP90AA1, HSP90AB1, TXN	5 (1468)
Gene expression (Transcription)	R-HSA-74160	HSP90AA1, SERPINE1, TXN, YWHAH, YWHAZ	5 (1851)
Metabolism	R-HSA-1430728	APOA1, CTSA, HSP90AA1, HSP90AB1, TXN	5 (3658)
Programmed Cell Death	R-HSA-5357801	HMGB1, HSP90AA1, YWHAH, YWHAZ	4 (218)
Cell Cycle	R-HSA-1640170	HSP90AA1, HSP90AB1, YWHAH, YWHAZ	4 (734)
Signalling by Interleukins	R-HSA-449147	CCL2, HMGB1, HSP90AA1, YWHAZ	4 (647)
Cytokine Signalling in Immune system	R-HSA-1280215	CCL2, HMGB1, HSP90AA1, YWHAZ	4 (1332)
Generic Transcription Pathway	R-HSA-212436	SERPINE1, TXN, YWHAH, YWHAZ	4 (1554)
RNA Polymerase II Transcription	R-HSA-73857	SERPINE1, TXN, YWHAH, YWHAZ	4 (1693)
TP53 Regulates Metabolic Genes	R-HSA-5628897	TXN, YWHAH, YWHAZ	3 (125)
HSP90 chaperone cycle for steroid hormone receptors (SHR)	R-HSA-3371497	HSP90AA1, HSP90AB1	2 (80)
Resistance of ERBB2 KD mutants to trastuzumab	R-HSA-9665233	HSP90AA1	1 (5)
Drug resistance in ERBB2 TMD/JMD mutants	R-HSA-9665737	HSP90AA1	1 (5)

## Data Availability

The mass spectrometry proteomics data have been deposited to the ProteomeXchange Consortium via the PRIDE partner repository with the dataset identifier PXD025556.

## Supplementary Materials

The following are available online at https://www.mdpi.com/article/10.3390/ijms222413297/s1.
