# Supplementary material for: Targeted Therapy Modulates the Secretome of Cancer-Associated Fibroblasts to Induce Resistance in HER2-Positive Breast Cancer
Source: Int J Mol Sci. 2021 Dec 10;22(24):13297. doi: 10.3390/ijms222413297 (PMC8706990; doi:10.3390/ijms222413297)
Supplement: Supplementary file 1 [file ijms-22-13297-s001.zip › ijms-1461130-supplementary.pdf]

Cytokine	Gene name	Position
Angiogenin	ANG	11D
BDNF	BDNF	6E
BLC (CXCL13)	CXCL13	7E
Ck beta 8-1 (CCL23)	CCL23	8E
EGF	EGF	9D
ENA-78 (CXCL5)	CXCL5	7A
Eotaxin-1 (CCL11)	CCL11	9E
Eotaxin-2 (CCL24)	CCL24	10E
Eotaxin-3 (CCL26)	CCL26	11E
FGF-4	FGF4	1F
FGF-6	FGF6	2F
FGF-7 (KGF)	FGF7	3F
FGF-9	FGF9	4F
FLT-3 Ligand	FLT3LG	5F
Fractalkine (CX3CL1)	CX3CL1	6F
G-CSF	CSF3	8A
GDNF	GDNF	8F
GM-CSF	CSF2	9A
GPC-2 (CXCL6)	CXCL6	7F
GRO a/b/g	CXCL1 / CXCL2 / CXCL3	10A
GRO alpha (CXCL1)	CXCL1	11A
HGF	HGF	9F
I-309 (CCL1)	CCL1	1B
IFN-gamma	IFNG	4C
IGF-1	IGF1	10D
IGFBP-1	IGFBP1	10F
IGFBP-2	IGFBP2	11F
IGFBP-3	IGFBP3	1G
IGFBP-4	IGFBP4	2G
IL-1 alpha (IL-1 F1)	IL1A	2B
IL-1 beta (IL-1 F2)	IL1B	3B
IL-10	IL10	11B
IL-12 (p40/p70)	IL12 (IL12B / IL12A)	1C
IL-13	IL13	2C
IL-15	IL15	3C
IL-16	IL16	3G
IL-2	IL2	4B
IL-3	IL3	5B
IL-4	IL4	6B
IL-5	IL5	7B

Cytokine	Gene name	Position
IL-6	IL6	8B
IL-7	IL7	9B
IL-8 (CXCL8)	CXCL8	10B
IP-10 (CXCL10)	CXCL10	4G
Leptina	LEP	5E
LIF	LIF	5G
LIGHT (TNFSF14)	TNFSF14	6G
MCP-1 (CCL2)	CCL2	5C
MCP-2 (CCL8)	CCL8	6C
MCP-3 (CCL7)	CCL7	7C
MCP-4 (CCL13)	CCL13	7G
M-CSF	CSF1	8C
MDC (CCL22)	CCL22	9C
MIF	MIF	8G
MIG (CXCL9)	CXCL9	10C
MIP-1 beta (CCL4)	CCL4	11C
MIP-1 delta	CCL15	1D
MIP-3 alpha (CCL20)	CCL20	9G
NAP-2 (CXCL7)	CXCL7	10G
NT-3	NTF3	11G
NT-4	NTF4	1H
OPG (TNFR SF 11)	TNFRSF11B	3H
OPN (SSP1)	SSP1	2H
OSM	OSM	1E
PARC	CCL18	4H
PDGF-BB	PDGFB	4E
PLGF	PGF	5H
RANTES (CCL5)	CCL5	2D
SCF	KITLG (SCF)	3D
SDF-1 alpha	CXCL12	4D
TARC (CCL17)	CCL17	5D
TGF beta 1	TGFB1	6D
TGF beta 2	TGFB2	6H
TGF beta 3	TGFB3	7H
TIMP-1	TIMP1	8H
TIMP-2	TIMP2	9H
TNF alpha	TNFA	7D
TNF beta (TNF SF 1B)	LTA (TNFB)	8D
TPO	TPO	2E
VEGF-A	VEGFA	3E

Supplementary Table S1. Map and antibody list. List of 80 cytokines analysed in the array and their positions.

GO biological process	cytokine count	mapped ID	genes	fold enrichment	FDR
regulation of immune system process (GO:0002682)	16	CCL2, CCL20, CCL4, CCL7, CCL8, CSF1, CXCL10, IGF1, IL5, IL7, LIF, MIF, TGFB1, TGFB2, THPO, TNFA	1701	7.45	2.53E-09
positive regulation of MAPK cascade (GO:0043410)	15	CCL15, CCL2, CCL20, CCL22, CCL26, CCL4, CCL7, CCL8, IGF1, LIF, MIF, TGFB1, TGFB2, THPO, TNFA	520	22.85	4.82E-15
regulation of cell motility (GO:2000145)	15	CCL2, CCL20, CCL26, CCL4, CCL7, CCL8, CSF1, CXCL10, FGF6, FGF7, IGF1, MIF, TGFB1, TGFB2, TNFA	947	12.55	1.43E-11
positive regulation of cellular metabolic process (GO:0031325)	14	ANG, CSF1, CXCL10, FGF6, FGF7, GDNF, IGF1, IL5, LIF, MIF, TGFB1, TGFB2, THPO, TNFA	3238	3.42	6.69E-04
regulation of protein phosphorylation (GO:0001932)	13	ANG, CSF1, FGF6, FGF7, IGF1, IL5, IL7, LIF, MIF, TGFB1, TGFB2, THPO, TNFA	1210	9.16	5.80E-09
neutrophil chemotaxis (GO:0030593)	13	CCL15, CCL2, CCL20, CCL22, CCL26, CCL4, CCL7, CCL8, CXCL1, CXCL10, CXCL2, CXCL3, TGFB2	77	> 100	6.61E-21
leukocyte chemotaxis (GO:0030595)	13	CCL15, CCL2, CCL20, CCL22, CCL26, CCL4, CCL7, CCL8, CXCL1, CXCL10, CXCL2, CXCL3, TGFB2	141	73.03	1.56E-18
lymphocyte chemotaxis (GO:0048247)	9	CCL15, CCL2, CCL20, CCL22, CCL26, CCL4, CCL7, CCL8, CXCL10	49	> 100	8.53E-15

GO molecular function	cytokine count	mapped ID	genes	fold enrichment	FDR
growth factor activity (GO:0008083)	13	CSF1, CXCL1, FGF6, FGF7, GDNF, IGF1, IL5, IL7, LIF, MIF,	163	58.32	3.96E-16

		TGFB1, TGFB2, THPO			
G protein-coupled receptor binding (GO:0001664)	12	CCL15, CCL2, CCL20, CCL22, CCL26, CCL4, CCL7, CCL8, CXCL1, CXCL10, CXCL2, CXCL3	294	32.33	3.20E-13
CCR chemokine receptor binding (GO:0048020)	8	CCL15, CCL2, CCL20, CCL22, CCL26, CCL4, CCL7, CCL8	45	> 100	6.56E-13
CCR2 chemokine receptor binding (GO:0031727)	2	CCL2, CCL7	5	> 100	7.63E-03

GO cellular component	cytokine count	mapped ID	genes	fold enrichment	FDR
extracellular space (GO:0005615)	26	ANG, CCL2, CCL4, CCL7, CCL8, CCL15, CCL20, CCL22, CCL26, CSF1, CXCL1, CXCL2, CXCL3, CXCL10, FGF6, FGF7, GDNF, IGF1, IL5, IL7, LIF, MIF, TGFB1, TGFB2, THPO, TNFA	3391	6.07	9.30E-18
secretory granule lumen (GO:0034774)	5	CXCL1, IGF1, MIF, TGFB1, TGFB2	321	12.34	3.19E-02
cytoplasmic vesicle lumen (GO:0060205)	5	CXCL1, IGF1, MIF, TGFB1, TGFB2	325	12.19	2.54E-02
platelet alpha granule lumen (GO:0031093)	3	IGF1, TGFB1, TGFB2	67	35.47	3.06E-02

Supplementary Table S2. GO enrichment analysis on gene sets generated from the list of 26 cytokines selected from the antibody-based array analysis. The “cytokine count” column indicates the number of cytokine genes assigned to the corresponding GO term.

pathway	count	p-value	gene ID
R-HSA-449147_Signaling by interleukins	14	0.0687	TNF, MIF, CCL20, TGFB1, CXCL1, CXCL2, CSF1, CCL22, CCL4, IL7, IL5, CCL2, CXCL10, LIF
R-HSA-6783783_Interleukin-10 signaling	10	0.0206	TNF, CCL20, CXCL1, CXCL2, CSF1, CCL22, CCL4, CCL2, CXCL10, LIF
Reactome_Interleukin-10 signaling	10	0.0206	TNF, CCL20, CXCL1, CXCL2, CSF1, CCL22, CCL4, CCL2, CXCL10, LIF
Boquest_Stem cell cultured vs fresh_up	9	0.0003	ANG, TGFB2, CXCL1, CXCL2, CXCL3, IGF1, CCL8, CCL2, CXCL10
Sana_TNF signaling_up	8	0.0062	CCL7, CCL20, CXCL2, CXCL3, CSF1, CCL8, CCL2, CXCL10
Lindstedt_Dendritic cell maturation A	8	0.0234	TNF, CCL20, CXCL1, CXCL2, CCL8, CCL4, CCL2, CXCL10
Phong_TNF response via p38 partial	6	0.0211	TGFB2, CCL20, CXCL1, CXCL2, CXCL3, LIF
KRAS600 lung breast up.V1_up	6	0.0910	CXCL2, CXCL3, LIF, CXCL1, CCL20, CCL22
Seki_Inflamatory response LPS_up	5	0.0094	CCL7, CSF1, CCL2, CXCL10, LIF
BMI1 down.V1_up	5	0.0094	TGFB2, CCL20, CXCL1, CSF1, LIF
STK33 up	5	0.0535	TNF, CXCL1, CXCL2, CXCL3, CCL4
EGFR up.V1_up	4	0.0289	TGFB2, CCL22, CCL2, LIF
MEL18 down.V1_up	4	0.0679	TGFB2, CXCL1, CSF1, LIF
R-HSA-202733_Cell surface interactions at the vascular wall	2	0.0910	MIF, TGFB1
MTOR up.V1_down	2	0.0910	CXCL1, IGF1
VEGFA up.V1_up	2	0.0910	CCL7, CCL2

Supplementary Table S3. Functional enrichment analysis of the selected 26 cytokines in the biological process category. The “count” column indicates the number of cytokine genes assigned to the corresponding GO term.

Pathway	Gene Set	p-value
KEGG_NOD_like_receptor_signalling _pathway	CCL2, IL6, HSP90B1, HSP90AB1, HSP90AA1	0.0007
Bystrykh_hematopoiesis_stem_cell_SCP2_qtl_trans	HSP90AB1, PSMD3, SPARC	0.0013
Gryder_PAX3FOXO1_top_enhancers	AP1B1, MCFD2, RCN1, YWHAZ, ACTG1, TBCA, UBAC2, GNL3L, PFDN4, GART, EWSR1, PXDN, NACA, APPL1	0.0014
Kyng_dna_damage_dn	ACTN4, PRKACA, CAB39, PRMT1, TPM1, FN1, SEMA3B, SERPINE2, PSG1, TBCA, LOX, NRP2	0.0024
Pid_PI3KCI_akt_pathway	PRKACA, YWHAH, YWHAZ, HSP90AA1	0.0038
Stearman_lung_cancer_early_vs_late_up	SF3B1, RCN1, ACTN4, PRMT1, NME3, HNRNPH1, EIF3M	0.0039
Pid_LKB1_pathway	PRKACA, CAB39, YWHAH, YWHAZ, HSP90AA1	0.0041
Cadwell_ATG16L1_targets_up	APOA1, BST1, HBA1, C1QTNF1, TF	0.0041
Faelt_b_cll_with_vh_rearrangements_up	NPC2, CAPZA2, HNRNPA3	0.0049
Altemeier_response_to_LPS_with_mechanical_ventilation	SERPINE1, CCL2, BST1, IL6, OSMR	0.0064
Turashvili_breast_lobular_carcinoma_vs_ductal_normal_up	COMP, FN1, POSTN, LOX, ADAM12, COL12A1, SPARC	0.0071
Croonquist_stromal_stimulation_up	SERPINE1, IL6, BASP1, IGFBP4, LOX, TMSB4X, SPARC	0.0071
PID_reg_gr_pathway	PRKACA, IL6, YWHAH, HSP90AA1, FGG	0.0095
Moreira_response_to_TSA_up	YWHAH, HSP90AB1, PA2G4, IGFBP4, TMSB4X	0.0095
Reactome_mTOR_signalling	CAB39, EIF4G1, CAB39L	0.0112
Reactome_interleukin_3_interleukin_5_and_GM_csf_signalling	PRKACA, YWHAZ, CRKL	0.0112
Xu_GH1_autocrine_targets_dn	TPM1, IGFBP4, TMSB4X, MPRIP, NLN, PXDN, COL12A1	0.0119
Biocarta_AKT_pathway	YWHAH, HSP90AA1	0.0121
Worschech_tumor_evasion_and_tolerogenicity_up	CCL2, IL6	0.0121
Turashvili_breast_lobular_carcinoma_vs_lobular_normal_dn	COMP, FN1, POSTN, LOX, ADAM12, COL12A1, NACA	0.0150

Supplementary Table S4. List of significant protein sets from CAF-200 secretome associated with biological reactions. As identified by enrichment analysis of differentially expressed proteins using the Molecular Signatures Database (MSigDB) of the GSEA software (Broad Institute).

A

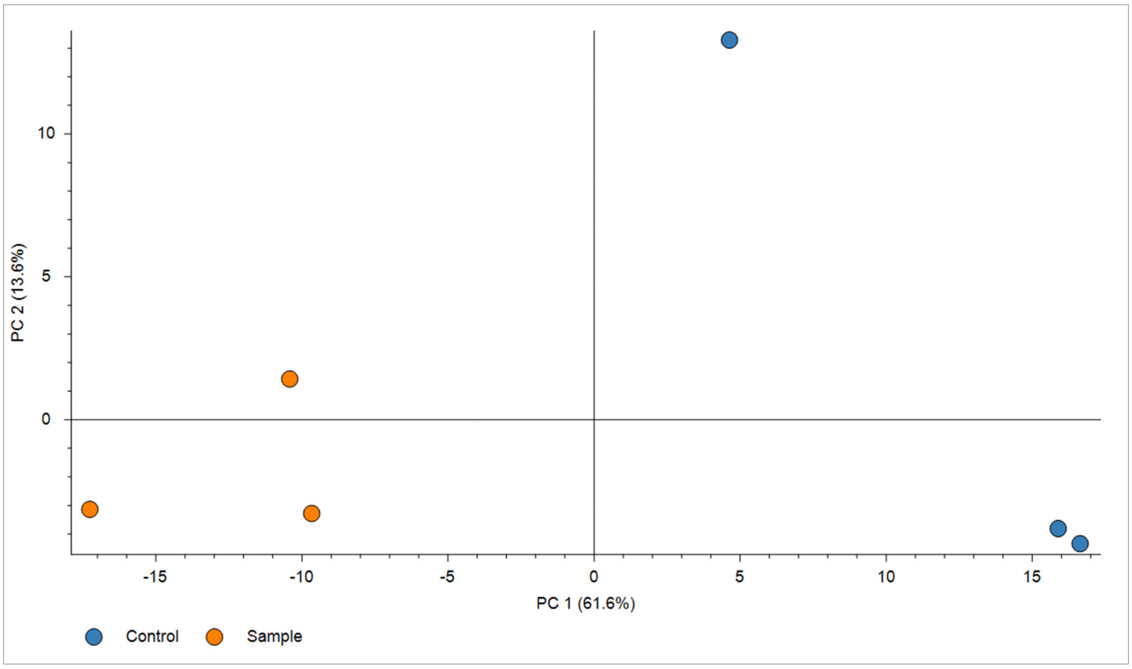

B

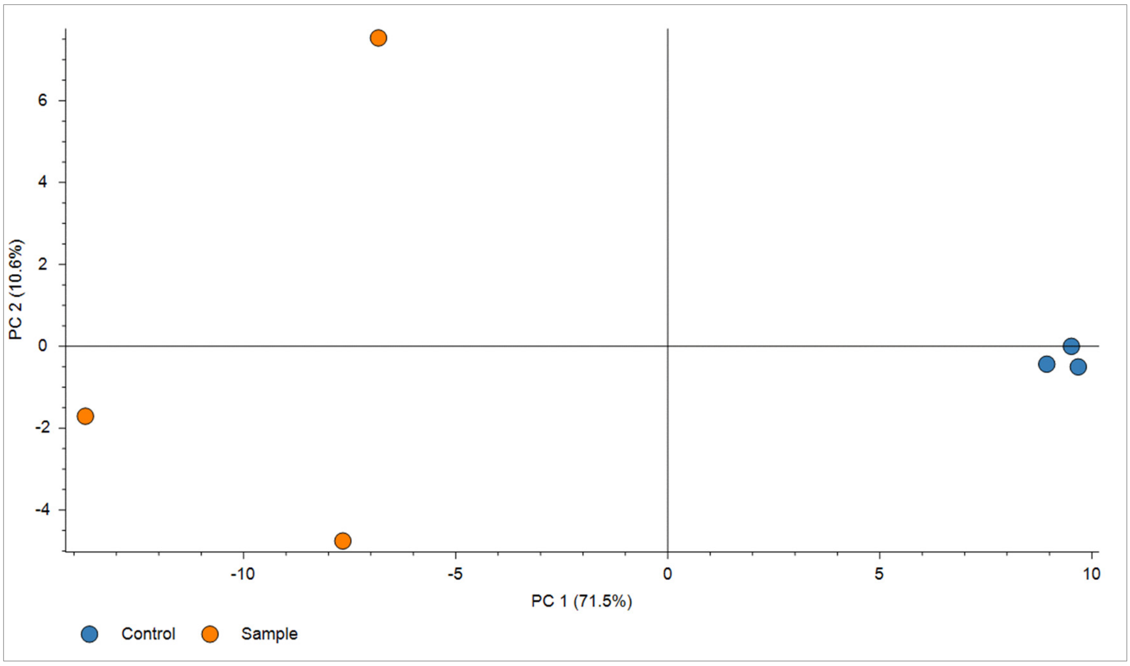

Supplementary Figure S1. Principal component analyses of CAF-200 secretome replicates: CAF-200 CM-control (non-treated) and CAF-200 CM-samples (treated with

trastuzumab plus pertuzumab plus docetaxel regimen) by LC-MS/MS analyses in pairwise comparisons. **A.** Graph PC1&PC2 shows grouping by condition treatment replicates and segregation among proteins identified with p-value<0.05. **B.** Graph PC1&PC2 shows grouping by condition treatment replicates and segregation among proteins with Abundance Ratio Variabilities = 0%.

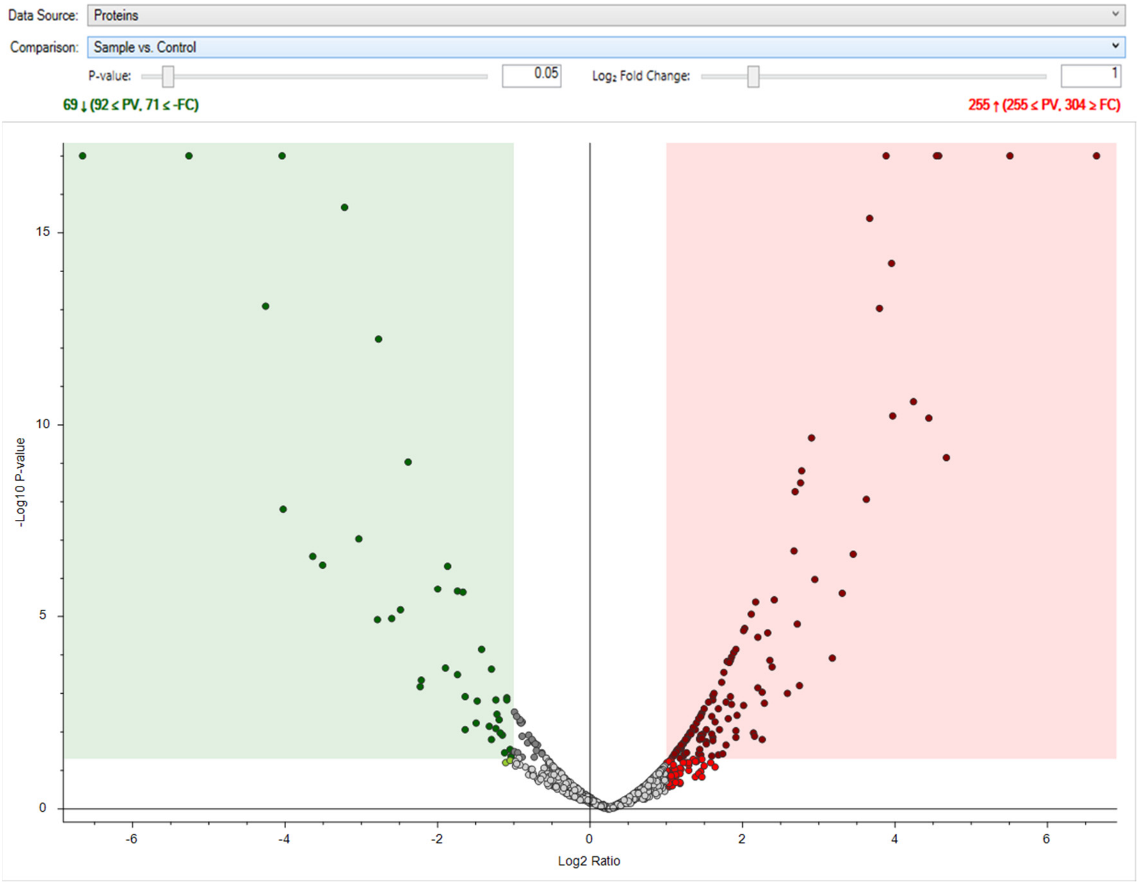

Supplementary Figure S2. Volcano plot analysis.

A

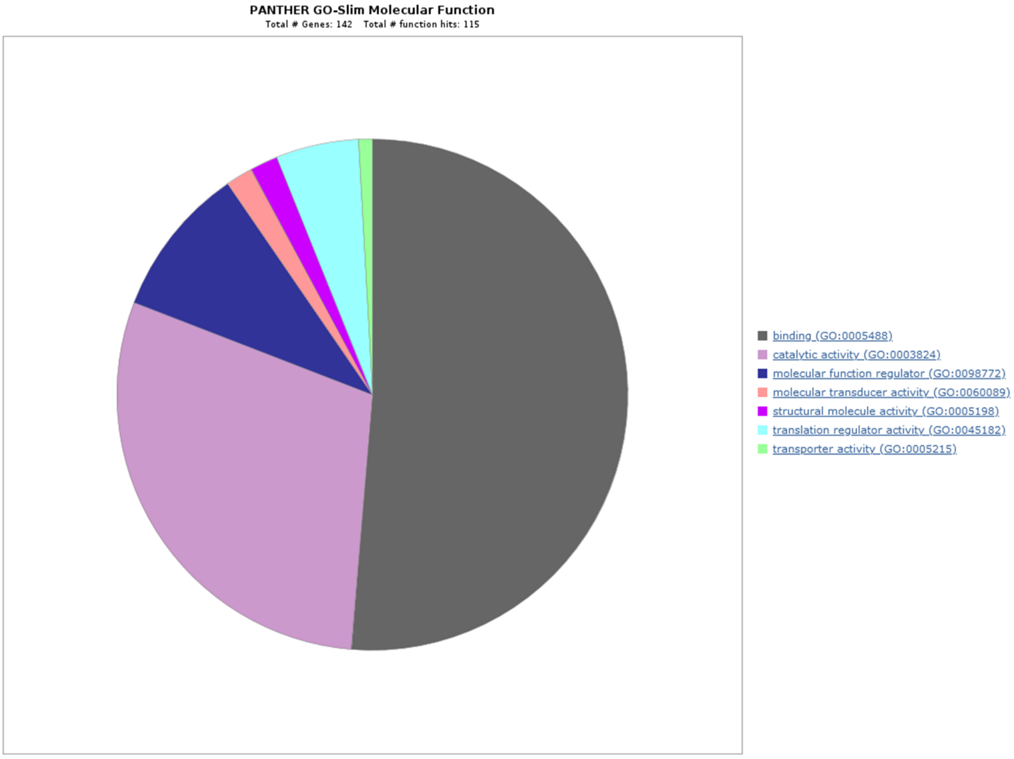
PANTHER GO-Slim Molecular Function
Total # Genes: 142 Total # function hits: 115
binding (GO:0005488)
catalytic activity (GO:0003824)
molecular function regulator (GO:0098772)
molecular transducer activity (GO:0060089)
structural molecule activity (GO:0005198)
translation regulator activity (GO:0045182)
transporter activity (GO:0005215)

B

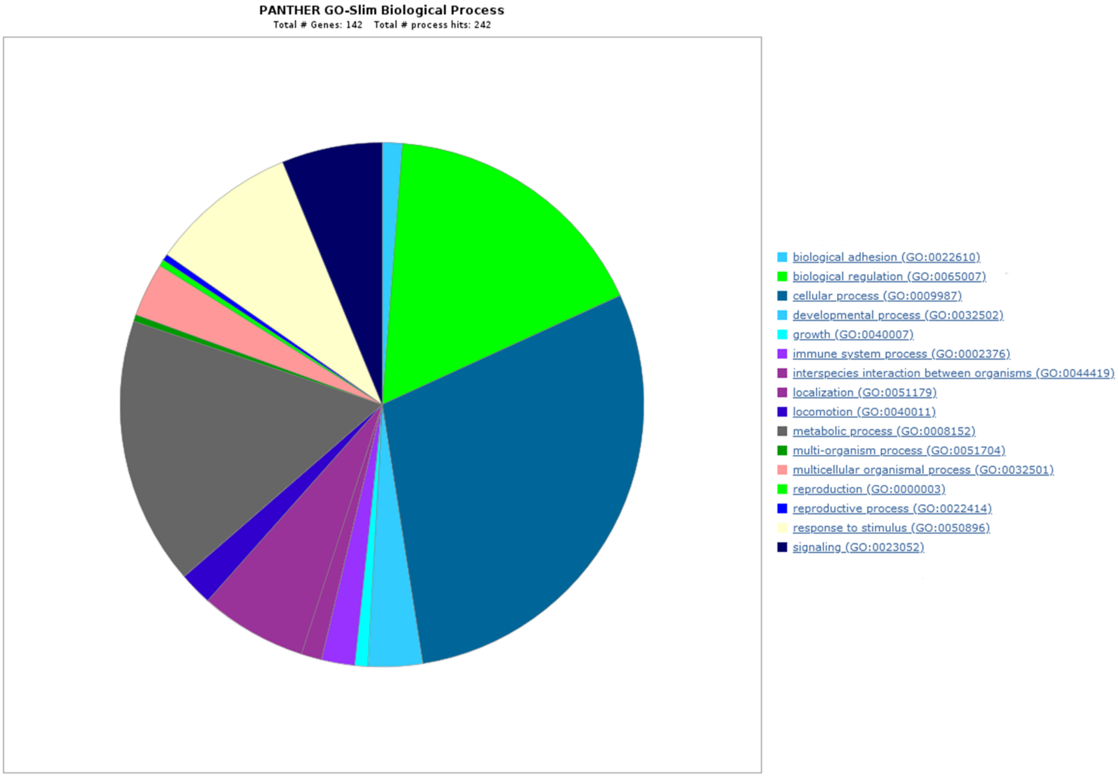
PANTHER GO-Slim Biological Process
Total # Genes: 142 Total # process hits: 242
biological adhesion (GO:0022610)
biological regulation (GO:0065007)
cellular process (GO:0009987)
developmental process (GO:0032502)
growth (GO:0040007)
immune system process (GO:0002376)
interspecies interaction between organisms (GO:0044419)
localization (GO:0051179)
locomotion (GO:0040011)
metabolic process (GO:0008152)
multi-organism process (GO:0051704)
multicellular organismal process (GO:0032501)
reproduction (GO:0000003)
reproductive process (GO:0022414)
response to stimulus (GO:0050896)
signaling (GO:0023052)

C

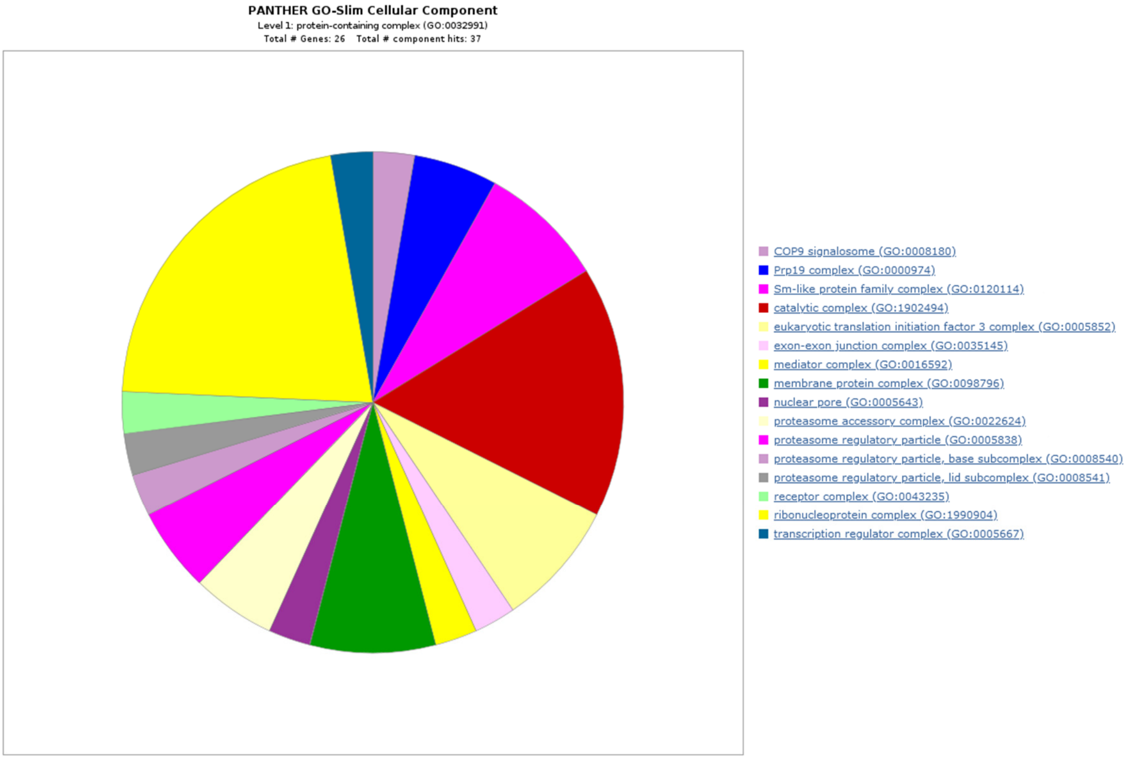
PANTHER GO-Slim Cellular Component
Level 1: protein-containing complex (GO:0032991)
Total # Genes: 26 Total # component hits: 37
COP9 signalosome (GO:0008180)
Prp19 complex (GO:0000974)
Sm-like protein family complex (GO:0120114)
catalytic complex (GO:1902494)
eukaryotic translation initiation factor 3 complex (GO:0005852)
exon-exon junction complex (GO:0035145)
mediator complex (GO:0016592)
membrane protein complex (GO:0098796)
nuclear pore (GO:0005643)
proteasome accessory complex (GO:0022624)
proteasome regulatory particle (GO:0005838)
proteasome regulatory particle, base subcomplex (GO:0008540)
proteasome regulatory particle, lid subcomplex (GO:0008541)
receptor complex (GO:0043235)
ribonucleoprotein complex (GO:1990904)
transcription regulator complex (GO:0005667)

D

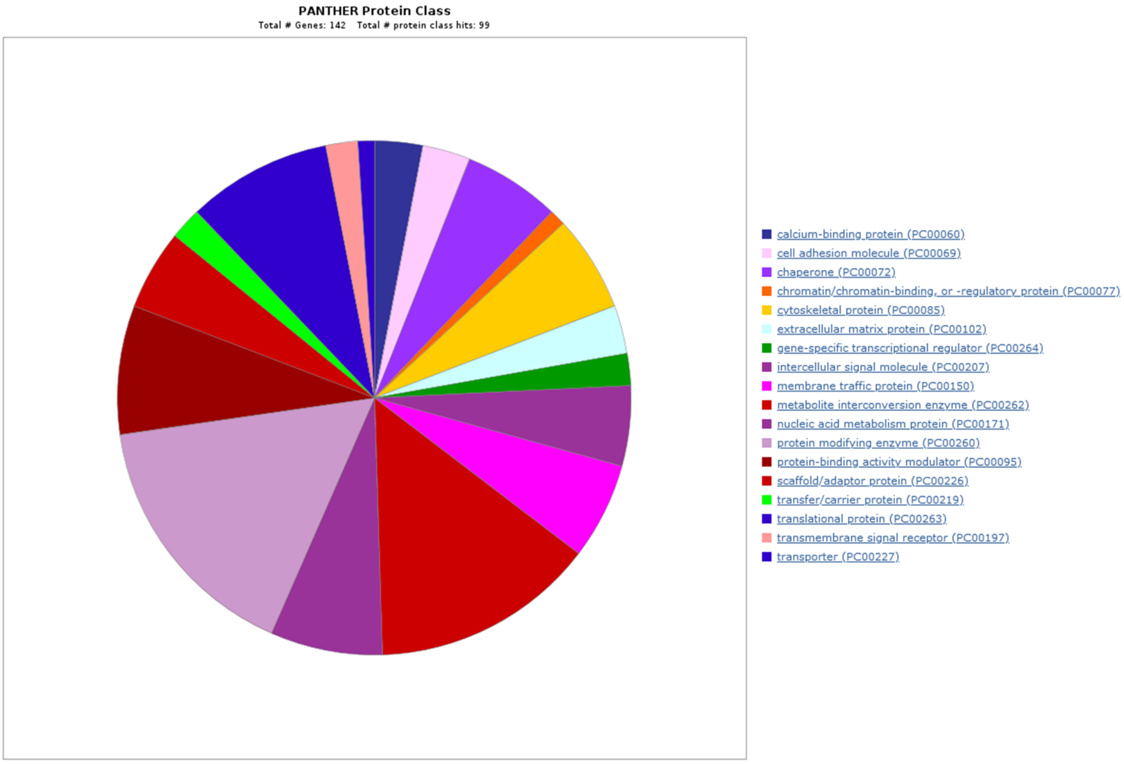
PANTHER Protein Class
Total # Genes: 142 Total # protein class hits: 99
calcium-binding protein (PC00060)
cell adhesion molecule (PC00069)
chaperone (PC00072)
chromatin/chromatin-binding, or -regulatory protein (PC00077)
cytoskeletal protein (PC00085)
extracellular matrix protein (PC00102)
gene-specific transcriptional regulator (PC00264)
intercellular signal molecule (PC00207)
membrane traffic protein (PC00150)
metabolite interconversion enzyme (PC00262)
nucleic acid metabolism protein (PC00171)
protein modifying enzyme (PC00260)
protein-binding activity modulator (PC00095)
scaffold/adaptor protein (PC00226)
transfer/carrier protein (PC00219)
translational protein (PC00263)
transmembrane signal receptor (PC00197)
transporter (PC00227)

E

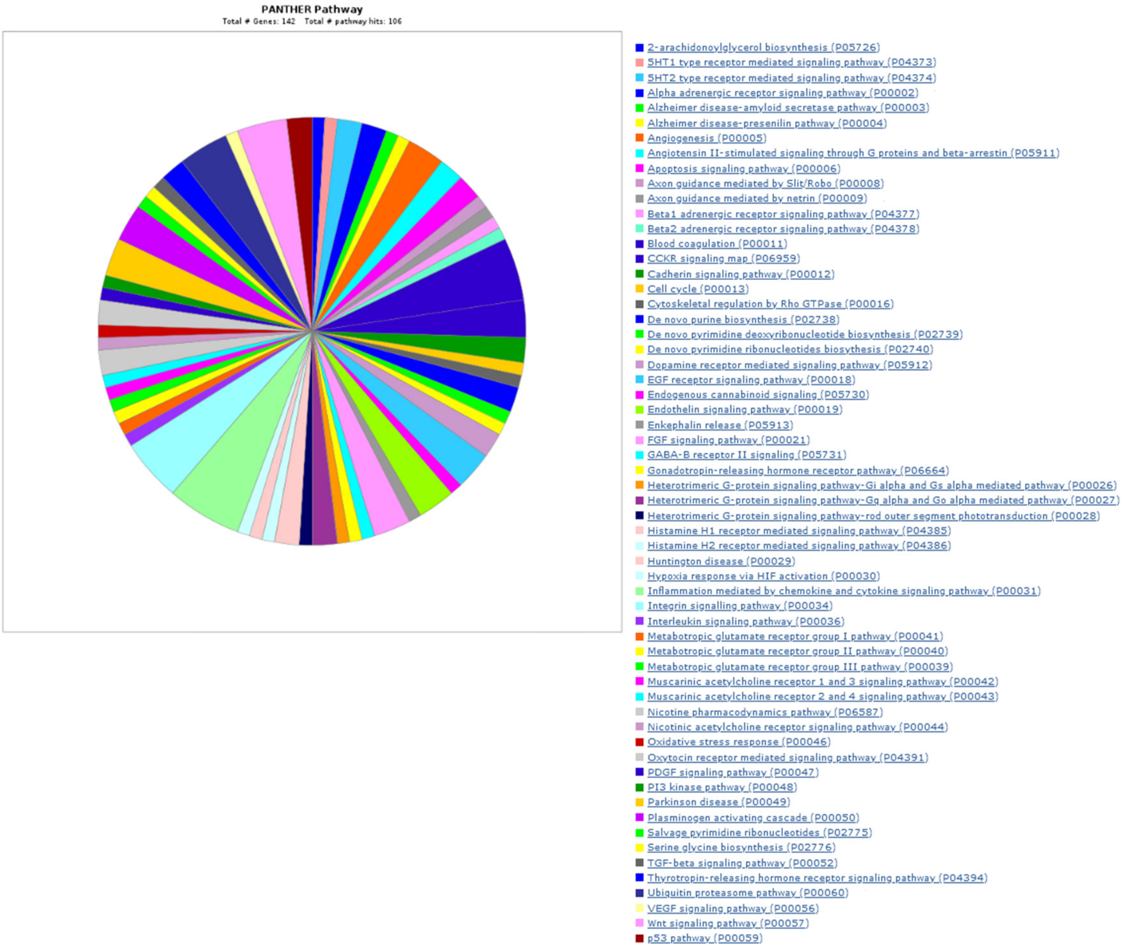

Supplementary Figure S3. GO-based functional annotation of candidate proteins. The list of 145 proteins was analysed for: **A**. Molecular function; **B.** Biological process; **C.** Cellular component; **D.** Protein class and **E.** Pathway.

A

B

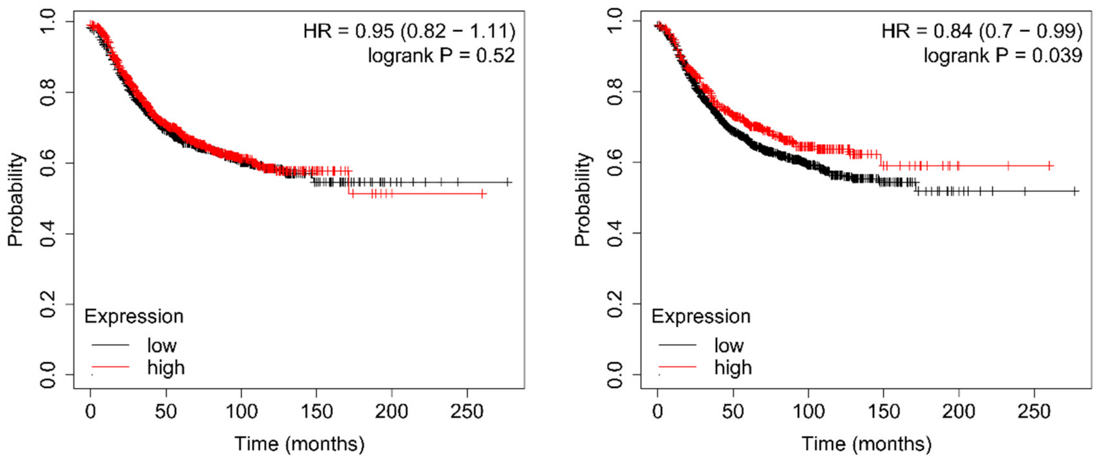

Supplementary Figure S4. RFS plots of molecular signatures assembled with: **A.** Eighty cytokines from the antibody arrays and **B.** Proteins with inverse abundance ratios from MS-MS discovery assays in the CAF-200 secretome.

A. Figure 2.

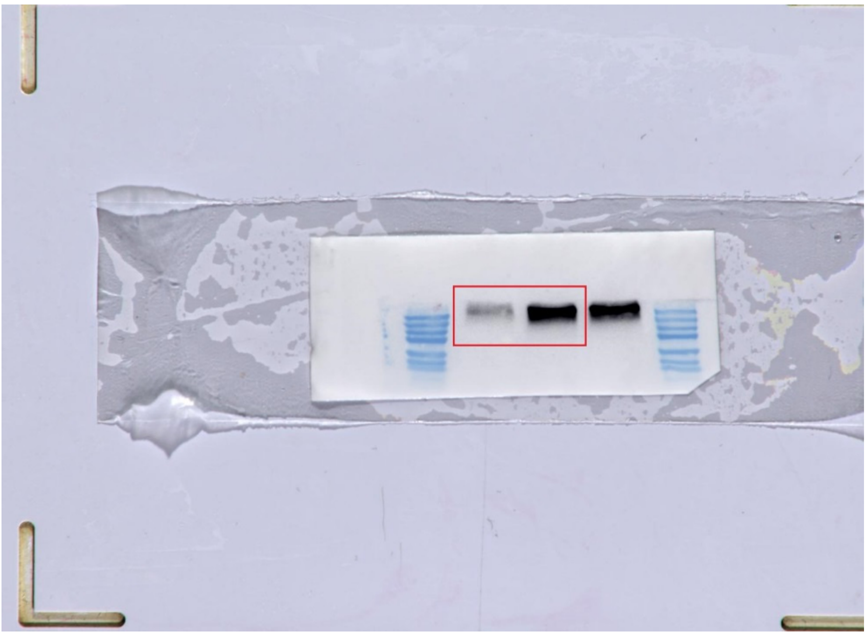

Fibronectin.

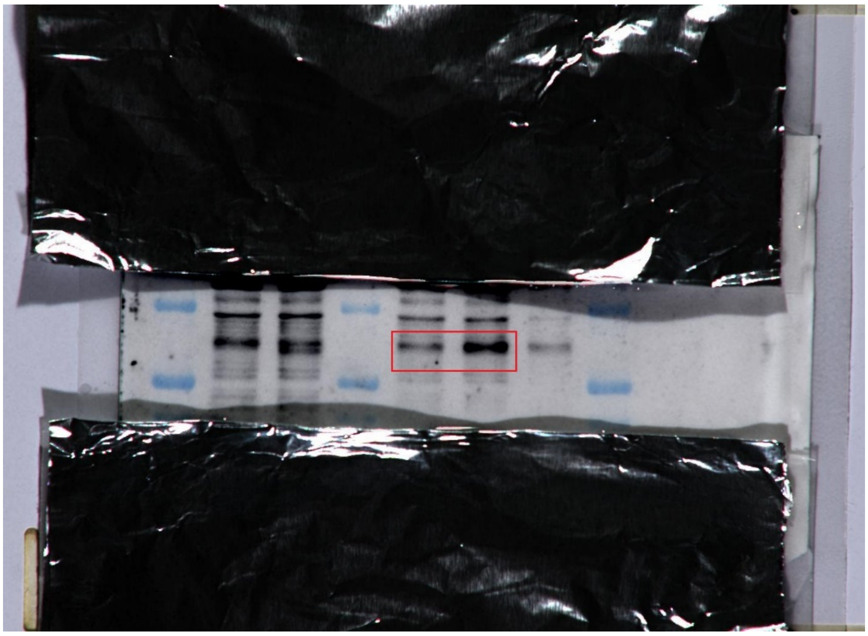

Snail.

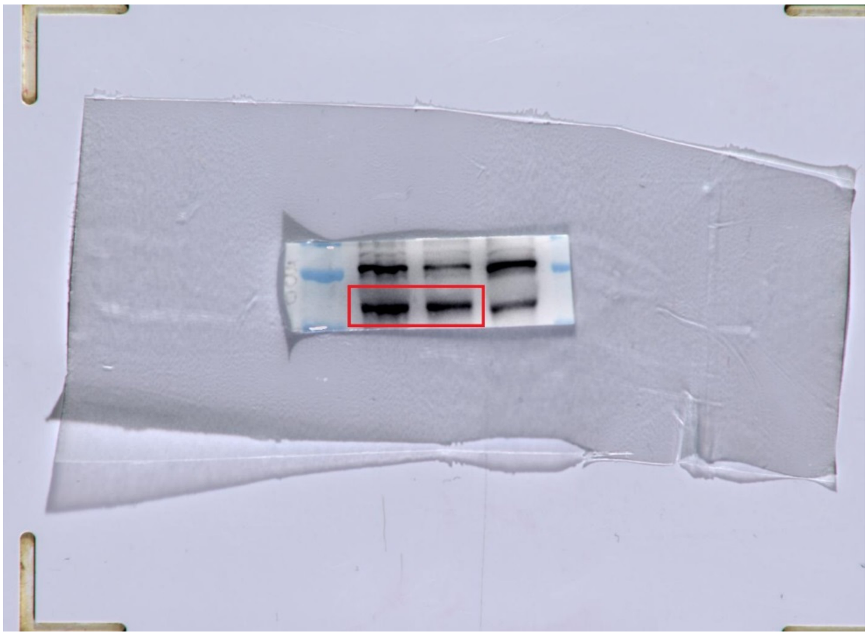

Occludin.

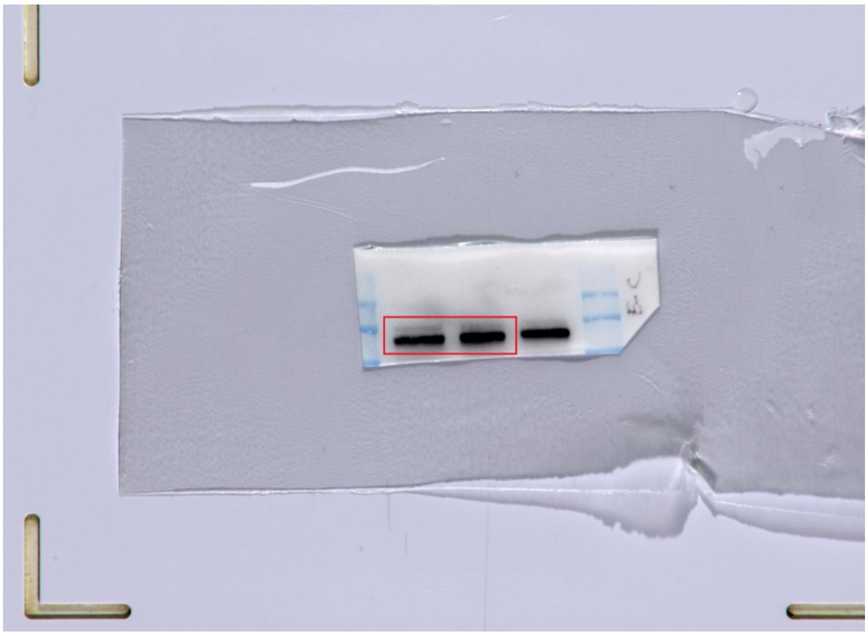

E-cadherin.

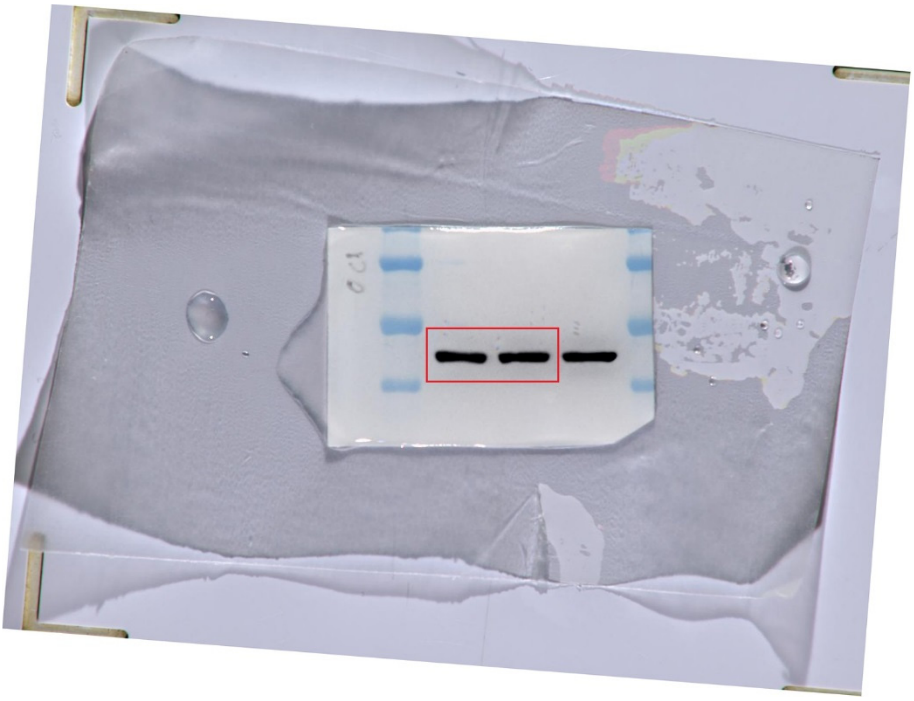

ß-actin.

B. Figure 3.

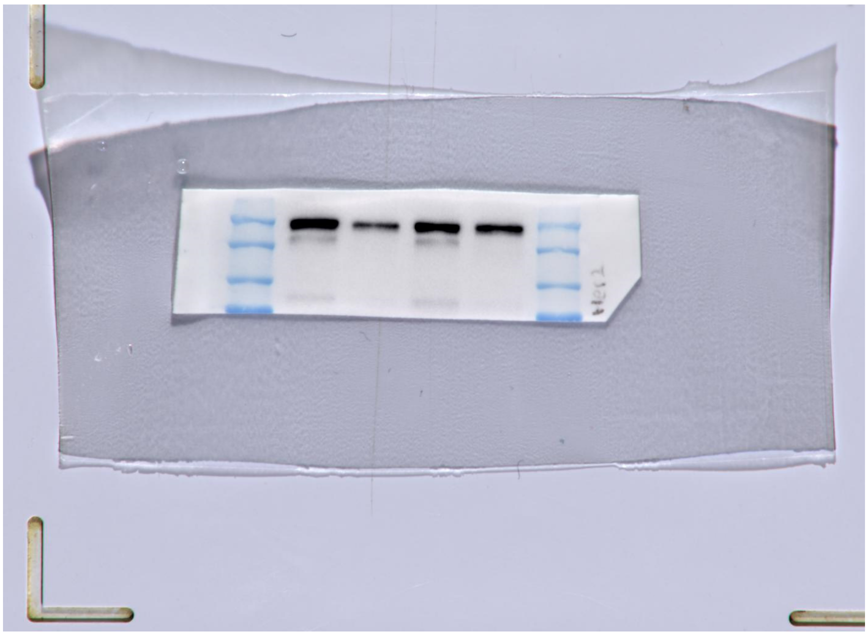

HER2.

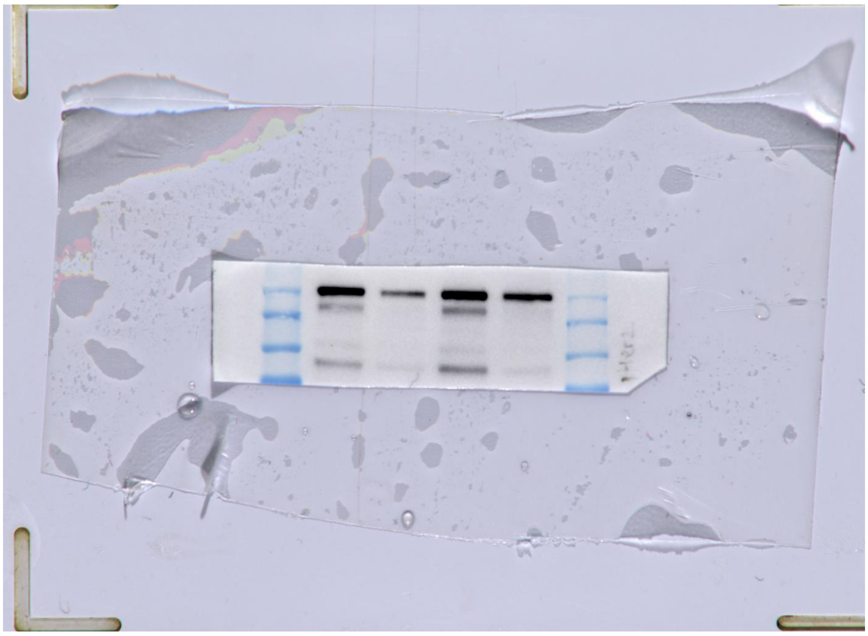

pHER2.

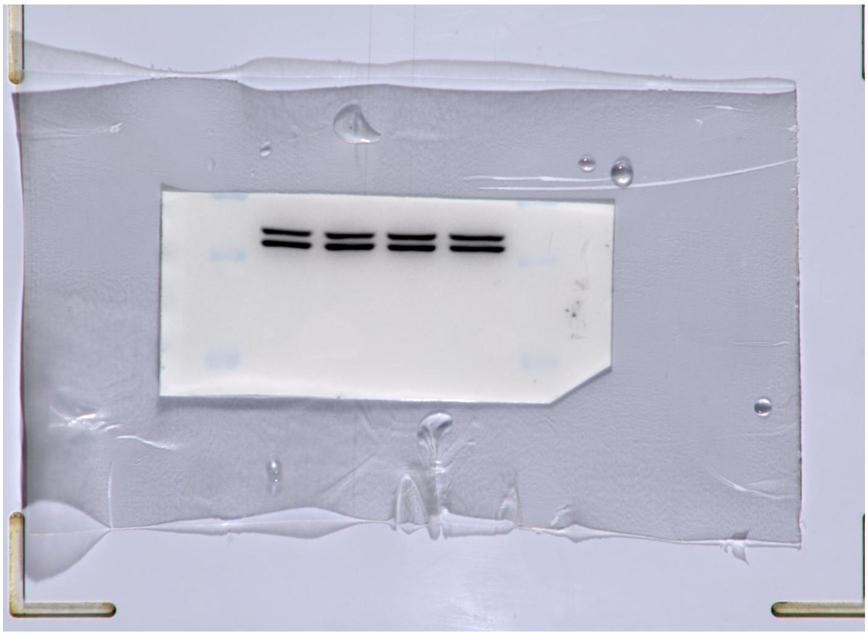

ERK.

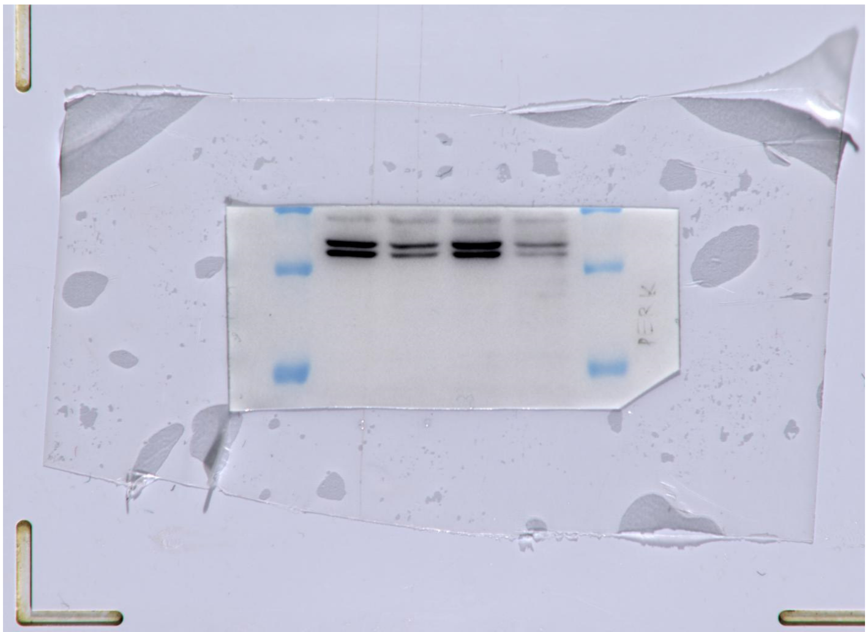

pERK.

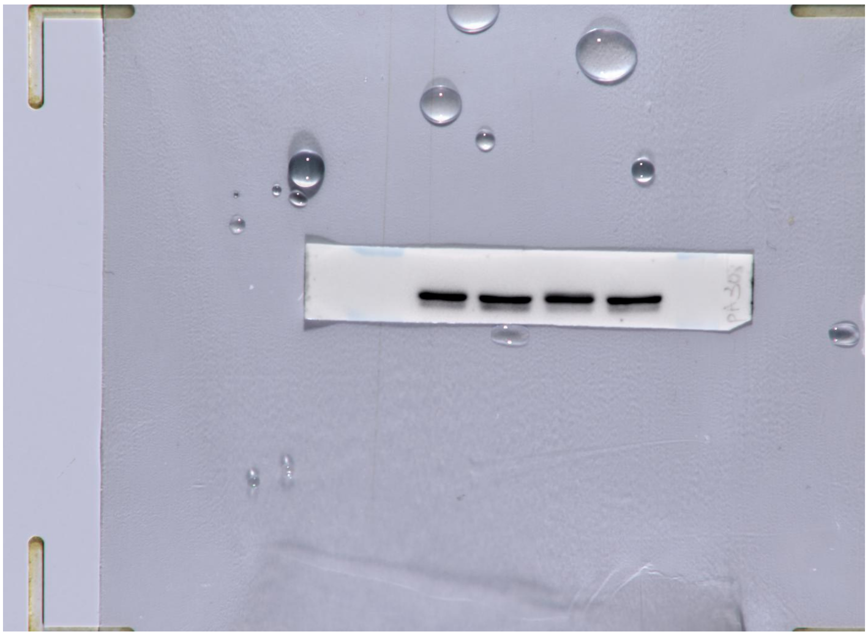

AKT.

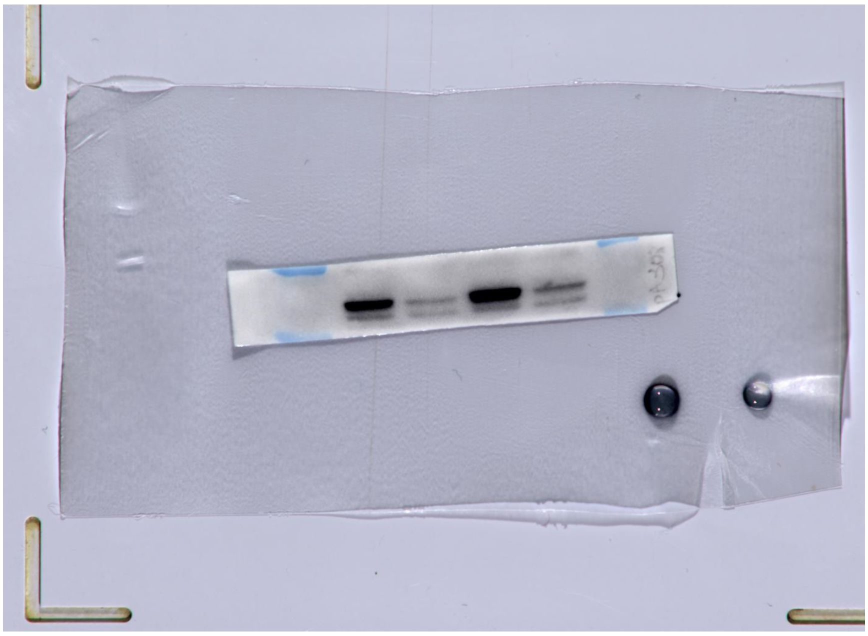

pAKT(Thr308).

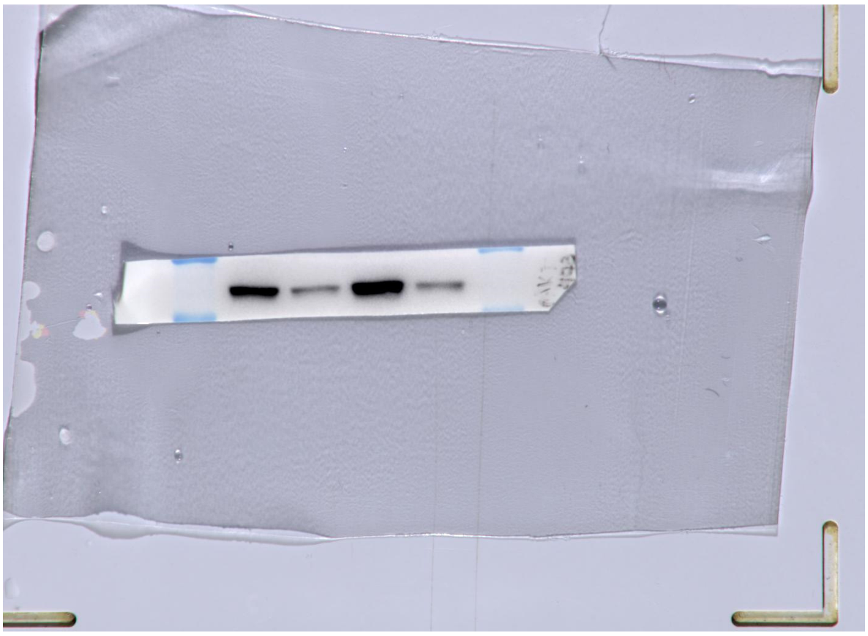

pAKT(Ser473).

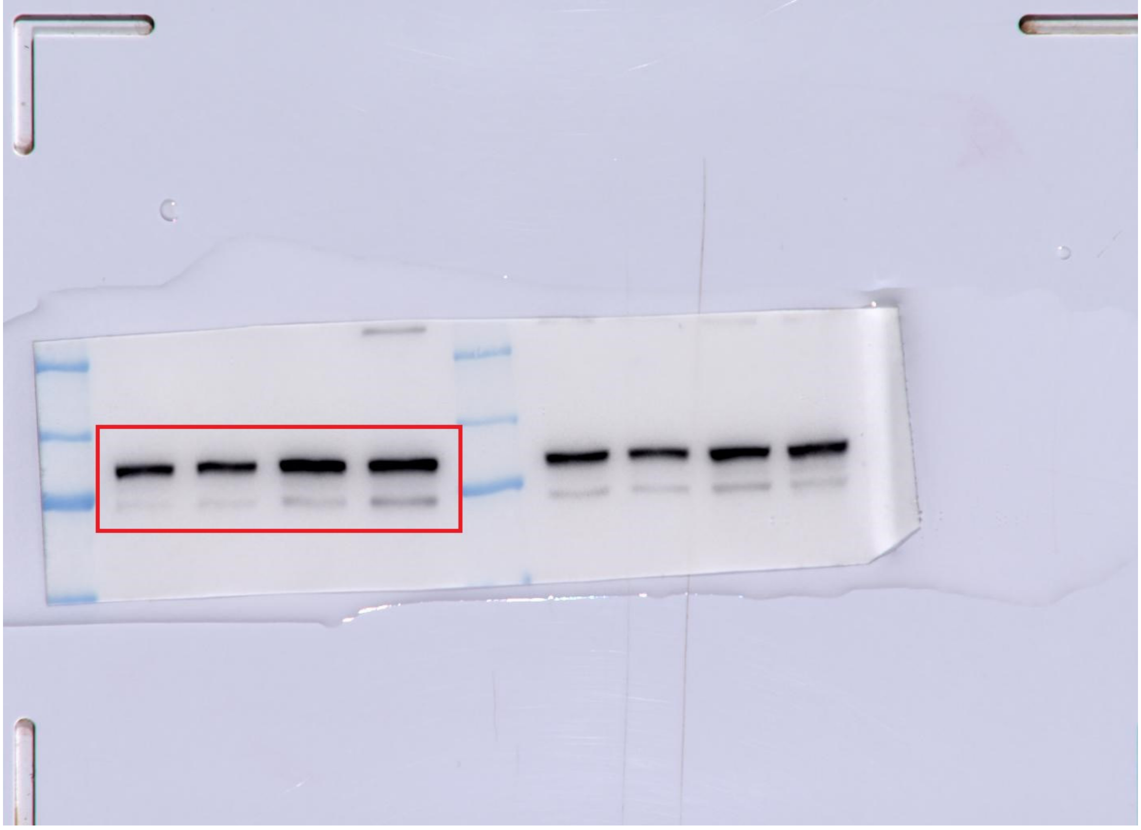

STAT3.

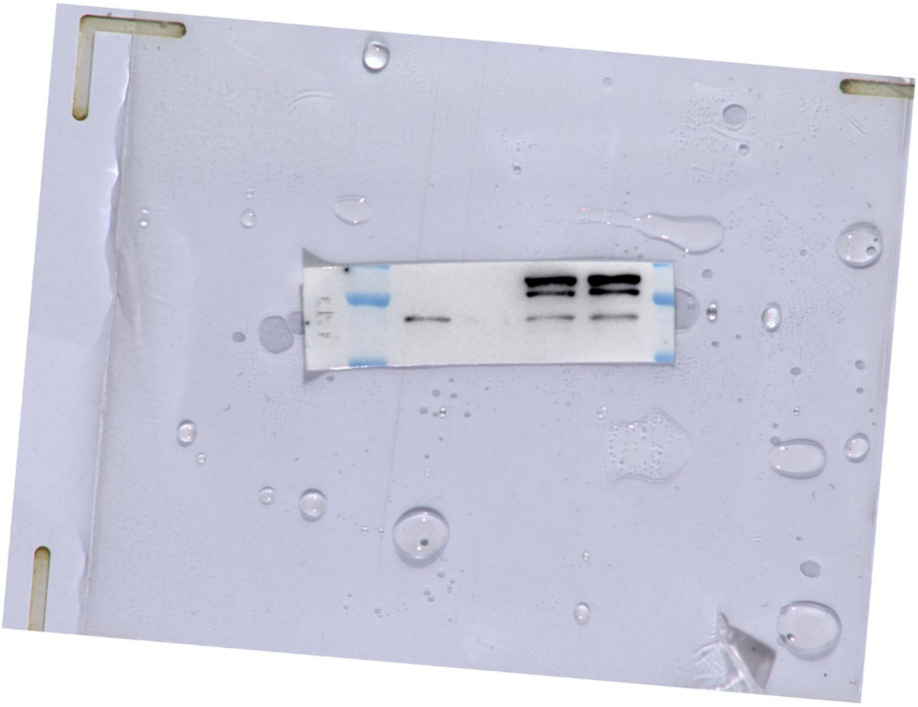

pSTAT3.

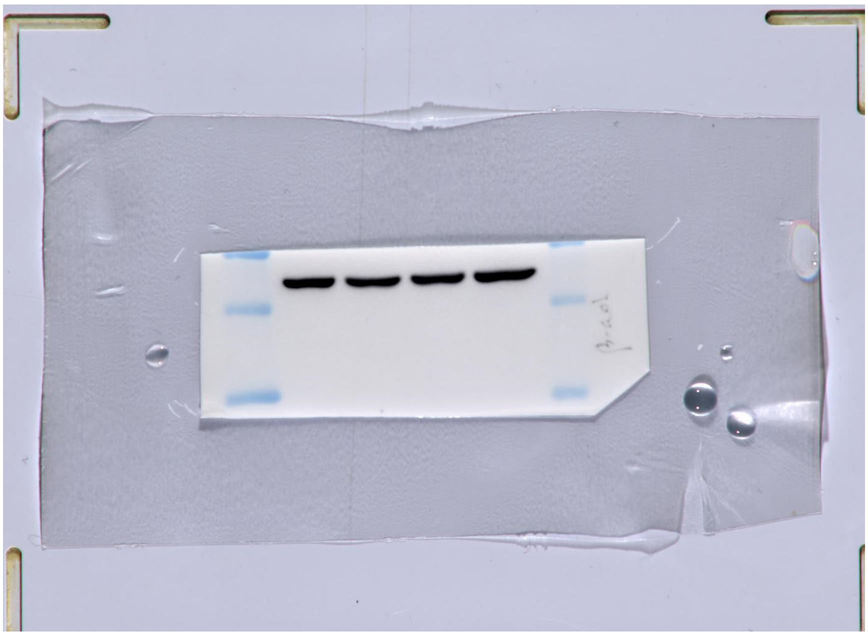

ß-actin.

Supplementary Figure S5. Uncropped WB images.
